# S- nitrosylation of Annexin A2 at Cys133 ameliorates pulmonary arterial hypertension by inhibiting the WNT/β-catenin pathway

**DOI:** 10.1186/s12931-025-03483-4

**Published:** 2026-03-18

**Authors:** Yu Wande, Chen Qianqian, Wang Yi, Zhu Minghui, Zhu Le, Gu Yue, Jiang Xiaomin, Wu Yichen, Luo Yang, Guanwen Ding, Zhang Hang

**Affiliations:** 1 Division of Cardiology, Nanjing First Hospital, Nanjing Medical University, 68 Changle Road, Nanjing, 210006 China; 2 3rd College, Nanjing Medical University, Nanjing, China; 3 3rd College, Kangda College of Nanjing Medical University, Nanjing, China; 4 2rd College, Emory University, Atlanta, USA

**Keywords:** Pulmonary arterial hypertension, ANXA2, S-nitrosylation, Pulmonary artery smooth muscle cells

## Abstract

**Background:**

The excessive proliferation and migration of pulmonary artery smooth muscle cells (PASMCs), which result in pulmonary vascular remodeling, are crucial pathological features of pulmonary arterial hypertension (PAH). Protein S-nitrosylation (SNO), which is a modification by which a nitric oxide (NO) group is added to a cysteine residue, has been shown to play a critical role in PASMC proliferation and PAH, but the underlying mechanism remains largely unknown.

**Methods:**

Using a combination of membrane and nuclear localization analyses, iodoTMT switch assays, molecular biology techniques, and in vitro and in vivo approaches, we investigated the effects of ANXA2 SNO at cysteine 133 (Cys133) on PASMC proliferation and migration in PAH.

**Results:**

ANXA2 protein expression was increased in PASMCs from rats with PAH. ANXA2 knockdown significantly inhibited excessive PASMC proliferation and migration. Additionally, inhibiting ANXA2 or conditionally knocking down ANXA2 in PASMCs ameliorated pulmonary vascular remodeling in experimental models of PAH. Moreover, we found that the NO donor S-nitrosoglutathione (GSNO) mediated the SNO of ANXA2 at Cys133 under hypoxic conditions. S-nitrosylated Cys133 (SNO-Cys133) of ANXA2 inhibited hypoxia-mediated PASMC proliferation and migration by regulating the subcellular localization of ANXA2 in cells. In addition, SNO-Cys133 of ANXA2 regulated the WNT pathway by inhibiting ANXA2 phosphorylation at Tyr24. Finally, SNO-Cys133 of ANXA2 ameliorated pulmonary vascular remodeling and improved RV function in vivo.

**Conclusions:**

SNO-Cys133 of ANXA2 suppressed ANXA2 relocation and the WNT pathway to ameliorate pulmonary vascular remodeling in PAH.

**Supplementary Information:**

The online version contains supplementary material available at 10.1186/s12931-025-03483-4.

## Introduction

Pulmonary arterial smooth muscle cells (PASMCs) play central roles in pathological vascular remodeling because, similar to cancer cells, they can excessively proliferate and migrate under hypoxic conditions [1]. Despite the use of combination therapy with multiple drugs, the long-term prognosis of pulmonary arterial hypertension (PAH) remains poor because of the complex pathogenic mechanism that leads to pulmonary small vessel remodeling [2, 3]. The molecular mechanisms underlying the contribution of PASMCs to PAH-associated pulmonary vascular lesions have been a major focus of research; however, these mechanisms currently remain unclear.

S-nitrosylation (SNO), which is a posttranslational modification by which nitric oxide (NO) is added to a cysteine residue, regulates protein activity, protein stability, and protein‒protein interactions [4]. Endothelial cell-derived NO enters PASMCs by diffusion and directly modifies G protein-coupled receptors through SNO, thus inhibiting vasoconstriction [5]. In addition, a previous study indicated that sodium nitrite reverses chronic hypoxic pulmonary hypertension in juvenile rats by increasing the SNO of proteins in the lungs [6]. However, the molecular mechanisms underlying protein SNO during the pathological progression of PAH have not been fully elucidated, even though the NO signaling pathway plays an important role in the pathological mechanism of PAH and is an important target for drug development.

ANXA2, which is a calcium ion-dependent phospholipid-binding protein, is widely distributed in the cytoplasm and membrane. The C-terminus of ANXA2 consists of four highly conserved and similar repetitive sequences, while the N-terminus contains multiple modification sites that regulate ANXA2 activity, including tyrosine 24 (Tyr24) and serine 25 (Ser25) phosphorylation sites, a Ser1 acetylation site, and S100A10 molecular binding sites [7, 8]. Notably, phosphorylation at Tyr24 plays a role in the membrane and nuclear translocation of ANXA2, thereby participating in the regulation of various biological cellular behaviors [9, 10]. Additionally, a previous study showed that the cysteine 133 (Cys133) site of ANXA2 is regulated by reversible glutathioneization, which inhibits the ability of ANXA2 to interact with phospholipid liposomes and F-actin [11]. However, the SNO of ANXA2 has not been evaluated until recently.

ANXA2 promotes cell proliferation, stabilizes the cytoskeleton, mediates endocytosis and exocytosis, and activates plasmin to dissolve the extracellular matrix by translocating to the cell membrane or nucleus [12, 13]. Bleomycin, which is a cytotoxic drug with side effects that lead to PAH, directly binds to ANXA2, resulting in pulmonary fibrosis. The genetic depletion of ANXA2 in mice mitigates bleomycin-induced pulmonary fibrosis [14]. In addition, recent findings have established that high ANXA2 expression plays an essential role in regulating the modulation of PASMC phenotypes during the pulmonary vascular remodeling that is associated with hepatopulmonary syndrome [15, 16]. Moreover, hypoxia-induced mitogenic factors promote PASMC migration by stimulating the translocation of S100A11 to the plasma membrane in PAH, and ANXA2 also synergistically translocates to the membrane [17]. These studies suggest that ANXA2 participates in regulating PAH development, but the specific mechanism has not been elucidated.

The WNT signaling pathway plays an important role in pulmonary vascular remodeling by increasing the proliferation and migration of PASMCs [18, 19]. Activation of the WNT signaling pathways depends on the nuclear translocation of β-catenin, which is a downstream effector [20]. Many studies have revealed that ANXA2 regulates the WNT signaling pathway to promote cell proliferation and migration during tumor development [21–23]. This phenomenon is related to the role of ANXA2 in promoting β-catenin translocation to the nucleus, and further studies have shown that ANXA2 may directly regulate β-catenin to increase the resistance of tumor cells [24].

In this study, we showed that ANXA2 levels were increased in PASMCs from PAH patients, and that ANXA2 participated in regulating the β-catenin-mediated downstream signaling pathway. More importantly, we demonstrated that the Cys133 site of ANXA2 undergoes SNO, which ameliorates PAH development by inhibiting ANXA2 phosphorylation at Tyr24.

## Methods

### Cell culture

Primary rat PASMCs were isolated from the pulmonary arteries normal rats or rats with PAH. Primary human PASMCs were obtained from the ScienCell Research Laboratory. Next, these cells were cultured in smooth muscle cell medium (ScienCell, Rockville, MD, USA) supplemented with fetal bovine serum (10%), 100 U/ml penicillin and 100 µg/ml streptomycin in the presence or absence of hypoxia (3% oxygen). PASMCs between passages 3 and 7 were used for experiments.

### Western blotting

PASMCs were lysed in lysis buffer (RIPA buffer supplemented with a protease inhibitor and phosphatase inhibitor cocktail), and total proteins were extracted (Beyotime, Shanghai, China). Surface and cytoplasmic proteins were extracted with a Surface and Cytoplasmic Protein Extraction Kit (P0033; Beyotime, Shanghai, China). Moreover, nuclear and cytoplasmic proteins were extracted with a Nuclear and Cytoplasmic Protein Extraction Kit (P0027; Beyotime, Shanghai, China). After the protein concentrations were measured, the proteins were separated by sodium dodecyl sulfate‒polyacrylamide gel electrophoresis and transferred to PVDF membranes. Primary anti-ANXA2 (1:1000 dilution; Cell Signaling Technology, Beverly, MA, USA), anti-β-catenin and anti-c-Myc antibodies (1:1000; Santa Cruz Biotechnology, CA) were used. An anti-GAPDH antibody (1:1000, BioWorld Technology, Tulare County, CA, USA) was used as the internal control for total and cytoplasmic proteins, an anti-Na-K-ATP antibody (1:1000; Cell Signaling Technology, Beverly, MA, USA) was used as the internal control for membrane proteins, and an anti-LaminB1 antibody (1:1000; Cell Signaling Technology, Beverly, MA, USA) was used as the internal control for nuclear proteins. The protein bands were imaged with a Syngene Bio Imaging Device (Syngene, Cambridge, UK), and the immunoreactive band density was analyzed with ImageJ software (National Institutes of Health, Bethesda, MD).

### Coimmunoprecipitation (Co-IP)

PASMCs were lysed with IP lysis buffer. After the protein concentrations were determined, equal amounts of total protein were incubated overnight with anti-ANXA2 or anti-β-catenin antibodies at 4 °C. Then, the immune complexes were mixed with protein A/G agarose beads (Thermo Fisher Scientific, MA, USA) at room temperature for 2 h to couple the antibodies to the agarose beads. Then, the agarose beads were washed with IP analysis buffer to obtain Co-IP products, which were subjected to Western blotting analysis.

### Detection of S-nitrosylated ANXA2 and mass spectrometry

The SNO of ANXA2 was assessed with iodoTMT Reagents (Thermo Fisher Scientific, MA, USA). The total proteins were extracted from cells, and then, the protein concentration was determined. Equivalent amounts of protein from each sample were blocked with 20 mM MMTS at room temperature for 30 min. Next, the MMTS was removed with acetone that had been prechilled at -20 °C. The proteins were resuspended in HENS buffer and incubated with iodoTMT Reagent, and then, sodium ascorbate was added and the mixture was incubated at room temperature for 2 h. The proteins were precipitated again with cold acetone and resuspended in HENS buffer. Subsequently, S-nitrosylated proteins were extracted by Co-IP with an anti-iodoTMT antibody. Finally, the S-nitrosylated proteins were separated by SDS‒PAGE and detected with an anti-ANXA2 antibody. For mass spectrometry analysis, trypsin was used to cleave total S-nitrosylated proteins into peptides, and these peptides were dissolved in TBS. The separated peptides were identified by liquid chromatography/tandem mass spectrometry (LC/MS/MS) and analyzed with an Orbitrap Exploris 480 instrument with a nanoelectrospray ion source. The precursor proteins and peptide fragments were analyzed with an Orbitrap detector.

### Small interfering RNA (siRNA) transfection and overexpression

PASMCs were plated in 6-well plates and cultured in fetal bovine serum-free Dulbecco’s modified Eagle’s medium. Then, the cells were transfected with the Lipofectamine™ 3000 transfection reagent (Invitrogen Corporation, Carlsbad, CA, USA). The cells in the control group were transfected with mimic NC as a negative control. The sense and antisense strands of the rat ANXA2-targeting siRNA were as follows: 5’-GAUGGUUCUGUUAUUGACUTT-3’ (sense) and 5’-AGUCAAUAACAGAACCAUCTT-3’ (antisense).

Cys133 and Tyr24 were each mutated to alanine (Ala). pcDNA3.1 plasmids carrying ANXA2, Cys133-mutant ANXA, or Cys133 + Tyr24-mutant ANXA, as well as the corresponding negative control (NC) plasmids, were obtained from Genecreate (Wuhan, China).

### Cell migration assay

Wound-healing and Transwell assays were performed to analyze PASMC migration. For the wound healing assay, PASMCs were seeded in 6-well plates, and a straight line was generated with a 200-µL pipette tip. The PASMCs were then cultured for an additional 24 h after the corresponding treatments. Images were captured with an optical microscope immediately after the wound was generated, and images were captured again after 12 and 24 h. In the Transwell assay, PASMCs were seeded with serum-free medium in the upper chambers of 24-well Transwell plates. After incubation for 24 h, the cells in the upper chambers were removed, and the cells on the lower surface of the insert were stained with 0.3% crystal violet for 15 min and then counted under an inverted microscope.

### Cell proliferation assay

Cell proliferation was performed using an EDU Cell Proliferation Assay kit (Beyotime, Shanghai, China) according to the manufacturer’s protocol.

### Immunofluorescence (IF)

After treatment, PASMCs and optimal cutting temperature compound-embedded lung tissue sections were fixed with 4% paraformaldehyde and permeabilized with 0.5% Triton X-100. After the samples were blocked with 5% bovine serum albumin for 1 h at room temperature, they were incubated overnight with primary antibodies at 4 °C and then exposed to anti-Rabbit/Mouse IgG (H + L) (1:100; Santa Cruz Biotechnology, Dallas, TX, USA). Images were captured by laser scanning confocal microscopy (LSM 710; Carl Zeiss, Germany).

### Experimental animals

To establish the PAH model, eight-week-old male C57BL/6J mice were housed under hypoxic conditions (10% O_2_) for four weeks and injected with SU5146 (20 mg/kg) weekly. In the second week of model establishment, six PAH model mice were subcutaneously injected with LCKLSL (1 mg/kg) daily. In addition, Tagln-Cre mice were transfected with a AAV9 virus carrying shANXA2 (rAAV Loxp-CMV-GFP-Loxp-shANXA2) via the tail vein. In the control group, Tagln-Cre mice were injected with AAV-Control. After 2 weeks of being housed under conditions that included a 12-hour light/dark cycle, periodic air changes, and free access to water and food, PAH was established in these mice. Using CRISPR-Cas9, the codon TGC (Cys) corresponding to amino acid 133 of ANXA2 was mutated to GCC (Ala) in C57BL/6 mice, and homozygous male mice carrying ANXA2 with mutant Cys133 were reared. Six C57BL/6J mice and six Cys133 mutant mice were administered SU5416 s.c. once a week during 4 weeks of exposure to chronic hypoxic conditions, and 1 mg/kg GSNO was intraperitoneally injected daily beginning on the 8th day of hypoxia.

At the end of model establishment, indicators of cardiac function, including the right ventricle internal dimension in diastole (RVID, d), the right ventricle anterior wall (RVAW) and the pulmonary artery acceleration time (PAT)-to-pulmonary artery ejection time (PET) ratio, were measured with a Vevo 2100 system (Fujifilm VisualSonics, Inc., Toronto, Canada) with a high-frequency (30 MHz) MS-400 transducer. Additionally, the right ventricular systolic pressure (RVSP) was determined with a pressure transducer catheter (Jinjiang Electronics, Sichuan, China). Lung tissues were embedded in paraffin and subjected to hematoxylin‒eosin (H&E) staining or embedded in optimal cutting temperature compound and subjected to IF staining. Right ventricular hypertrophy was determined with the following formula: right ventricle/(septum + left ventricle).

All the animal experiments were performed in accordance with the Guide for the Care and Use of Laboratory Animals (National Research Council), and this study was approved by the Institutional Animal Care and Use Committee of Nanjing Medical University (DWSY-23078382, Nanjing, China).

### RNA sequencing and analysis

Rat PASMCs that were transfected with siANXA2 were subjected to hypoxic conditions for 24 h, whereas those in the control group were subjected to hypoxic conditions alone. Then, RNA was isolated from these cells via TRIzol extraction (Sigma-Aldrich, USA). A poly-A-selected RNAseq library was prepared with an Illumina mRNA Preparation Kit (Illumina, CA, USA) and sequenced by Illumina NovaSeq. In addition, RNA sequencing data (GSE72181) of 3 hypoxia group and 3 normoxia group were downloaded from the GEO database. Differentially expressed genes were identified on the basis of an adjusted p value threshold of 0.05 and a log2-fold change of +/-1.5. The functional enrichment analysis of the overlapping differentially expressed genes was conducted with the Kyoto Encyclopedia of Genes and Genomes (KEGG) database.

### Statistical analysis

All the data are presented as the means ± standard deviations (SDs). T tests and one-way analysis of variance (ANOVA) were performed with SPSS 22.0 software (version 24.0). All the tests were two-sided, and a p value < 0.05 was considered to indicate a statistically significant difference.

## Results

### ANXA2 knockdown suppressed the proliferation and migration of PASMCs from PAH rats

**Fig. 1:**
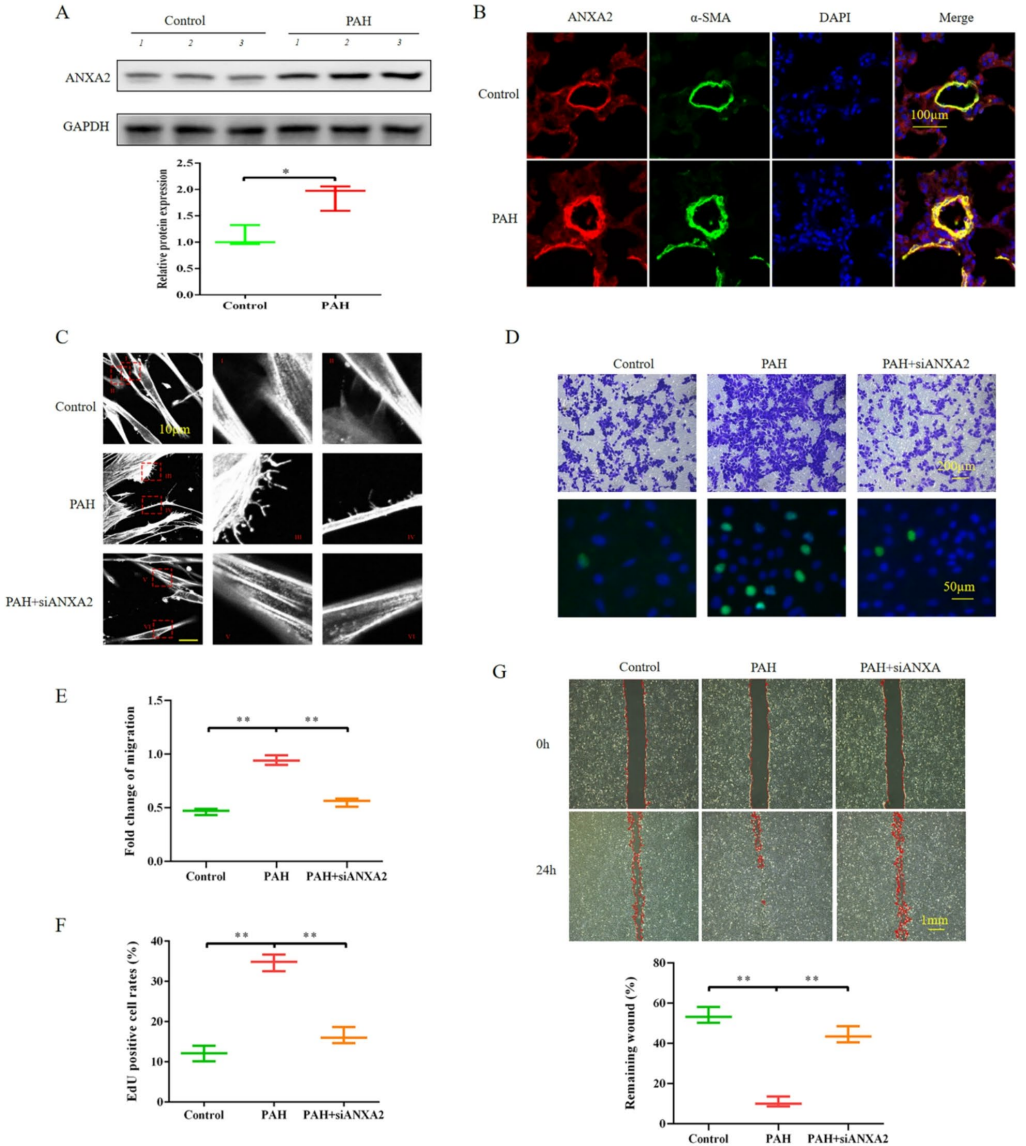
ANXA2 knockdown alleviated the proliferation and migration of PASMCs from rats with PAH. **A** Representative Western blotting images and quantified densitometry ratios of ANXA2 expression in lung tissues from rats with PAH (*n* = 3) and control rats without PAH (*n* = 3). **B** Representative confocal immunofluorescence images of pulmonary tissues from rats with PAH and control rats without PAH stained with antibodies against ANXA2 (red), antibodies against alpha-SMA (green) and DAPI (blue). Scale bar = 100 μm. **C** PASMCs were transfected with ANXA2 siRNA or NC siRNA for 24 h, and the F-actin antibody-stained pseudopodia of human PASMCs were observed by confocal microscopy. Scale bar = 10 μm. **D**, **G** After ANXA2 knockdown for 24 h, cell proliferation and migration were determined by Transwell (up of **D**, scale bar = 200 μm), EdU (drown of **D**, scale bar = 50 μm) and wound healing(**G**, scale bar = 100 μm) assays. **E** Statistical analysis of the fold change of migration. **F** Statistical analysis of the number of EdU-positive cells. The results are presented as the means ± SDs; *, *p* < 0.05; **, *p* < 0.01

To explore the role of ANXA2 in PASMCs in the context of PAH, we examined the ANXA2 levels in PASMCs from rats with PAH or control rats. Compared with those in control rats without PAH, the ANXA2 protein levels were increased in PASMCs from rats with PAH (Fig. 1A-B). We further assessed the functional consequences of ANXA2 knockdown in PASMCs from rats with PAH. ANXA2 knockdown significantly inhibited the formation of pseudopodia in PASMCs from rats with PAH (Fig. 1C). Moreover, Transwell assays and wound healing revealed that ANXA2 knockdown suppressed the migration of PASMCs from rats with PAH compared with the scramble sequence (Fig. 1D, E and G). Moreover, ANXA2 knockdown inhibited the proliferation of PASMCs from rats with PAH, as shown by the EdU incorporation assay (Fig. 1D and F). Additionally, inhibition of ANXA2 via LCKLSL treatment for 24 h suppressed wound healing and the number of migrating cells among PASMCs that were isolated from PAH patients (Figure S1A-S1C). These results suggest that increased ANXA2 expression may contribute to pulmonary vascular remodeling by promoting excessive cell proliferation and migration.

### Inhibition of ANXA2 mitigated pulmonary vascular remodeling and improved right ventricular function

**Fig. 2:**
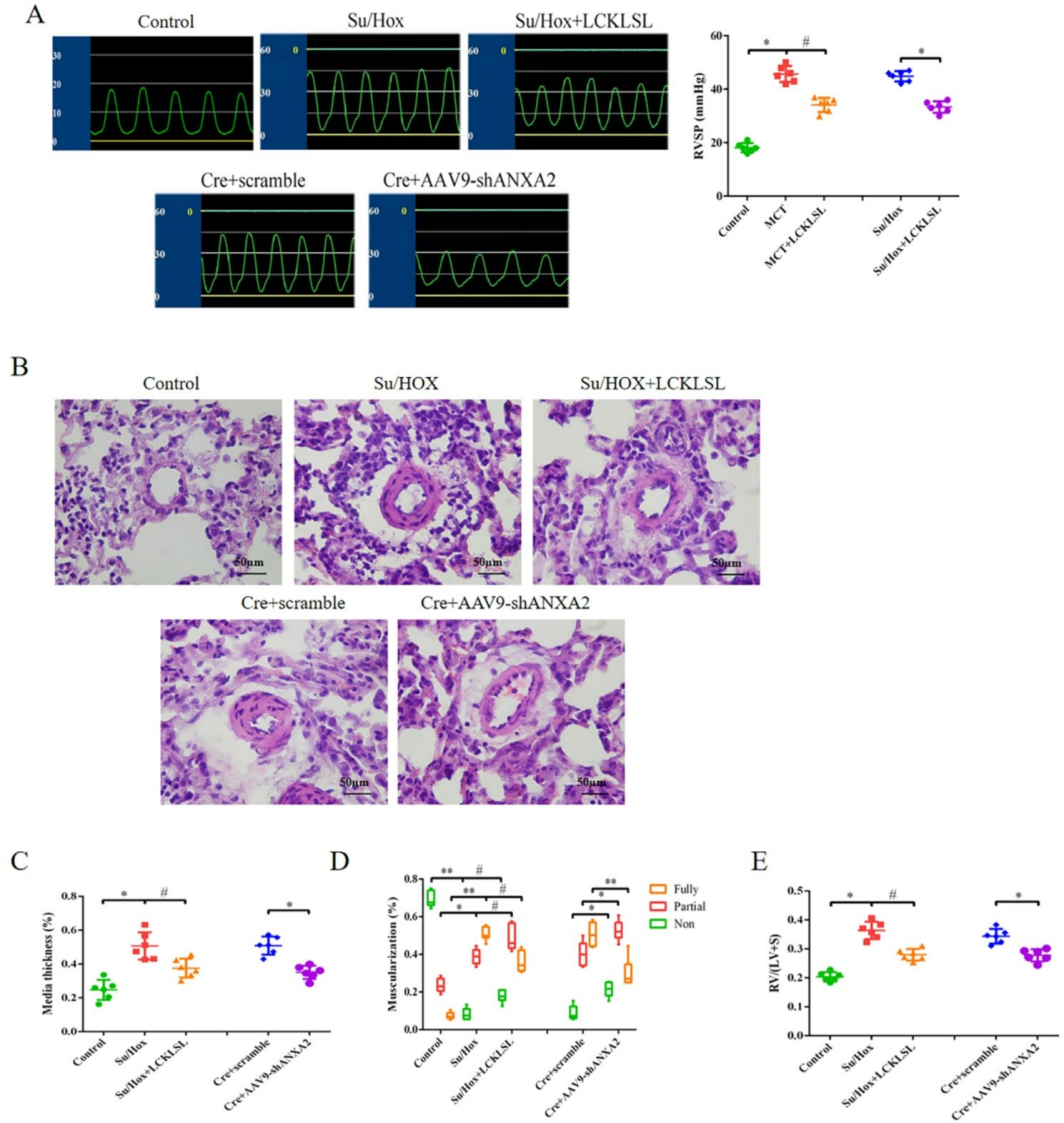
ANXA2 knockdown mitigated pulmonary vascular remodeling and improved right ventricular function. Eight-week-old mice were exposed to hypoxic conditions (10% O_2_) for 4 weeks and injected with SU5416 once a week. In the last two weeks, the mice continuously received 5 mg/day/kg body weight PX-12 via subcutaneous injection every day. In addition, 6-week-old Tagln-Cre mice were injected with AAV9-EGFP-shANXA2 via the caudal vein to generate smooth muscle cell-specific conditional ANXA2-knockdown mice. After 2 weeks, all the transgenic mice were exposed to hypoxic conditions (10% O_2_) for 4 weeks. **A** Comparison of RVSP as measured by a right heart catheter in each group. **B** Representative images of HE-stained distal pulmonary arterioles reflecting pulmonary vascular remodeling. Scale bar = 50 μm. **C**-**E** Comparison of the medial wall thickness (**C**), percentage of muscularization (**D**) and RV/(LV + S) weight (**E**) in each group. The results are presented as the means ± SDs; *n* = 6 per group. *, *p* < 0.05; **, *p* < 0.01; ^#^, *p* < 0.05; ^##^, *p* < 0.01

We next investigated the effect of ANXA2 on the development of PAH. Experimental rats and mice with PAH were treated with the ANXA2 inhibitor LCKLSL. In addition, an AAV9 vector carrying Cre-dependent short hairpin ANXA2 (shANXA2) was injected into Tagln-Cre transgenic mice to conditionally knockdown ANXA2 in smooth muscle cells, and these smooth muscle cells were to investigate the role of ANXA2 in SU5416/hypoxia (Su/Hox)-induced PAH. ANXA2 inhibition or conditional knockdown significantly inhibited the Su/Hox-induced increase in RVSP in PAH model mice (Fig. 2A). In addition, the Su/Hox-induced aberrant pulmonary vascular remodeling and right ventricular remodeling were partly reversed by treatment with an ANXA2 inhibitor for 2 weeks or conditional ANXA2 knockdown (Fig. 2B and E). Moreover, the ANXA2 inhibitor decreased the pulmonary artery pressure and alleviated the aberrant pulmonary vascular remodeling and right ventricular remodeling in PAH model rats induced by MCT or Su/Hox (Figure S2A-S2E). Thus, inhibiting ANXA2 is a novel potential strategy for the prevention and treatment of hypoxia-induced PH.

### S- nitrosylation of Cys133 alleviated PASMC proliferation and migration by reversing ANXA2 translocation

**Fig. 3:**
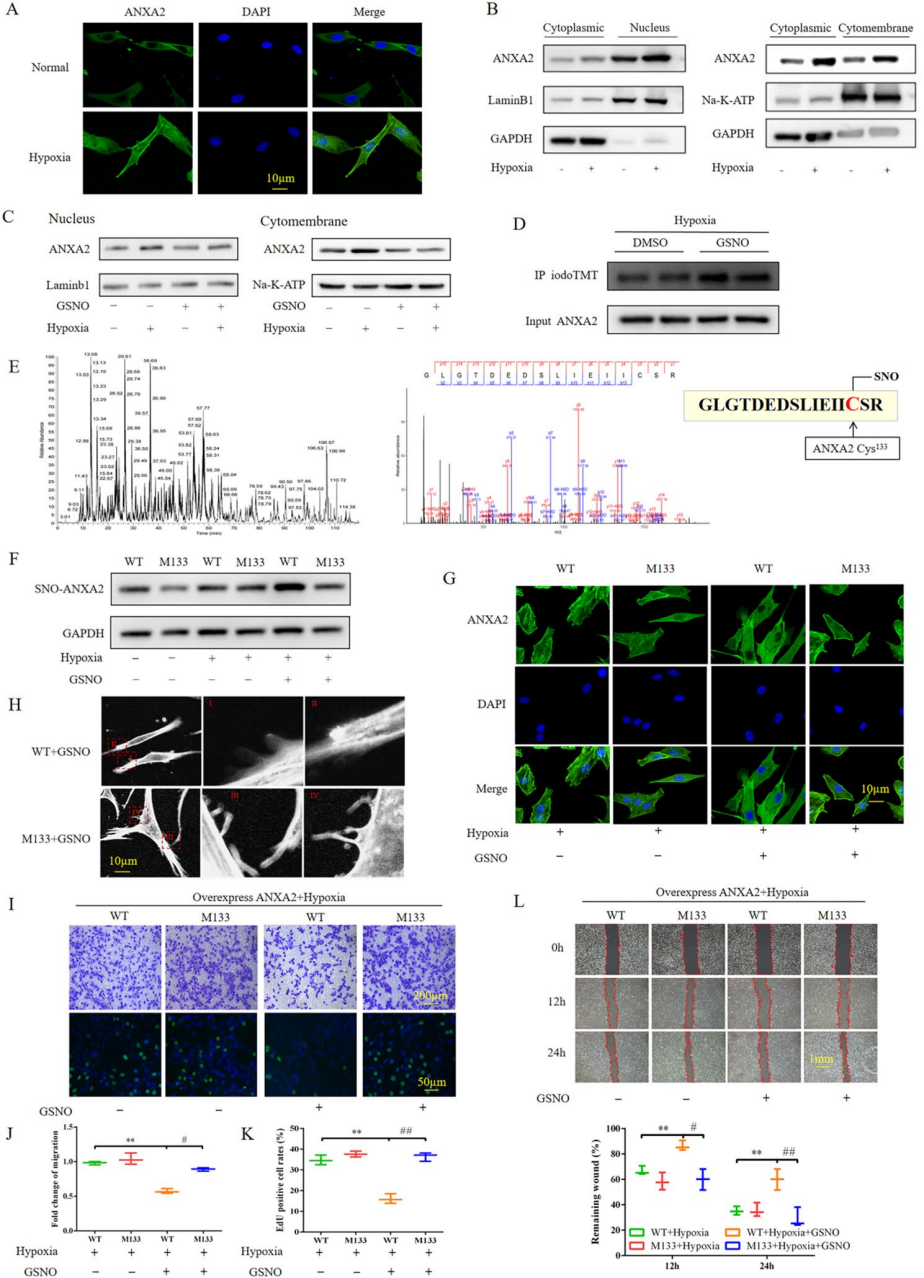
nitrosylation of Cys133 alleviated PASMC proliferation and migration by reversing ANXA2 translocation. **A** Representative confocal immunofluorescence images of the intracellular location of ANXA2 in human PASMCs after 24 h of exposure to hypoxia. Scale bar = 10 μm. **B** Immunoblotting analysis of ANXA2 expression in the membrane, cytoplasm, and nucleus of rat PASMCs after 24 h of exposure to hypoxia. **C** Immunoblotting analysis of ANXA2 expression in the membrane, cytoplasm, nucleus of human PASMCs after 2 h of GSNO treatment under hypoxic conditions (3% oxygen for 24 h). **D** Immunoblotting analysis of iodoTMT reagent-modified ANXA2 expression in human PASMCs after 2 h of GSNO treatment under hypoxic conditions. The ANXA2 control was obtained from immunoblotting of PASMC lysate. **E** Base peak ion chromatograms of total SNO protein in human PASMCs after 24 h of exposure to hypoxic conditions (left). The base peak chromatogram of the peptide containing the Cys133 site of ANXA2 (right). **F** Western blotting analysis of the levels of SNO of ANXA2 with Cys133 mutation and wild-type ANXA2 under normoxic conditions, hypoxic conditions, and hypoxic conditions with GSNO treatment, respectively. **H** The Cys133 mutant plasmid or wild-type plasmid was transfected into human PASMCs for 24 h, the cells were then treated with GSNO under hypoxic conditions for 2 h, and the F-actin antibody-stained pseudopodia of human PASMCs were observed by confocal microscopy. Scale bar = 10 μm. **G**, **I**, **L** Rat PASMCs overexpressing Cys133-mutant or wild-type ANXA2 were treated with GSNO for 2 h under hypoxic conditions. The intracellular location of ANXA2 in PASMCs was observed by laser scanning confocal microscopy (**G**, scale bar = 10 μm). Cell proliferation and migration were determined by Transwell (up of **I**, scale bar = 200 μm), EdU incorporation assays (drown of **I**, scale bar = 50 μm) and wound healing (**L**, scale bar = 400 μm). **J** Statistical analysis of the fold change of migration. **K** Statistical analysis of the number of EdU-positive cells. The results are presented as the means ± SDs; *, *p* < 0.05; **, *p* < 0.01

The membrane and nuclear translocation of ANXA2 are important mechanisms underlying cellular morphological and functional changes [25, 26]. Therefore, we performed IF staining to determine the intracellular distribution of ANXA2. After exposure to hypoxic conditions, ANXA2 significantly translocated to the membrane and nucleus (Fig. 3A). Immunoblotting analysis further confirmed that ANXA2 levels were increased in the membrane and nucleus of human PASMCs after hypoxic stimulation and that the total protein level of ANXA2 was increased (Fig. 3B). NO signaling plays a major role in modulating vascular tone and remodeling in pulmonary circulation [27]. To investigate whether ANXA2 expression is regulated by the NO signaling pathway, we treated human PASMCs with S-nitrosoglutathione (GSNO) after exposure to hypoxia. First, GSNO significantly reduced the membrane and nuclear translocation of ANXA2 in cells exposed to hypoxic conditions for 24 h (Fig. 3C). Additionally, the NO-induced SNO of proteins is important for their biological activities. Interestingly, the SNO of ANXA2 increased after GSNO treatment (Fig. 3D). Subsequently, an idoTMT-switch assay combined with liquid chromatography‒tandem mass spectrometry revealed that Cys133 was the site of ANXA2 that was modified by SNO after treatment with GSNO under hypoxic conditions (Fig. 3E). Thus, we replaced the Cys133 of ANXA2 with alanine and transfected a plasmid encoding this mutant into PASMCs. Cys133 mutation significantly inhibited the GSNO-induced SNO of ANXA2 under hypoxic conditions but did not affect the SNO of ANXA2 under baseline conditions or hypoxic conditions alone (Fig. 3F). Moreover, IF analysis revealed that GSNO did not suppress the translocation of Cys133-mutant ANXA2 to the cell membrane or nucleus (Fig. 3G). In addition, Cys133 mutation promoted the formation of pseudopodia in PASMCs, even when these cells were treated with GSNO under hypoxic conditions (Fig. 3H). Furthermore, Transwell, Edu incorporation assay and scratch wound assays revealed that GSNO suppressed the hypoxia-induced proliferation and migration of human PASMCs, whereas Cys133 mutation completely altered this phenomenon (Fig. 3I and L). Taken together, our results indicate that SNO of ANXA2 at Cys133 ameliorated the excessive proliferation and migration of PASMCs in the context of PAH by reducing ANXA2 membrane and nuclear translocation under hypoxic conditions.

### SNO of Annexin A2 at Cys133 in PASMCs inhibited hypoxia-induced WNT pathway activation

**Fig. 4:**
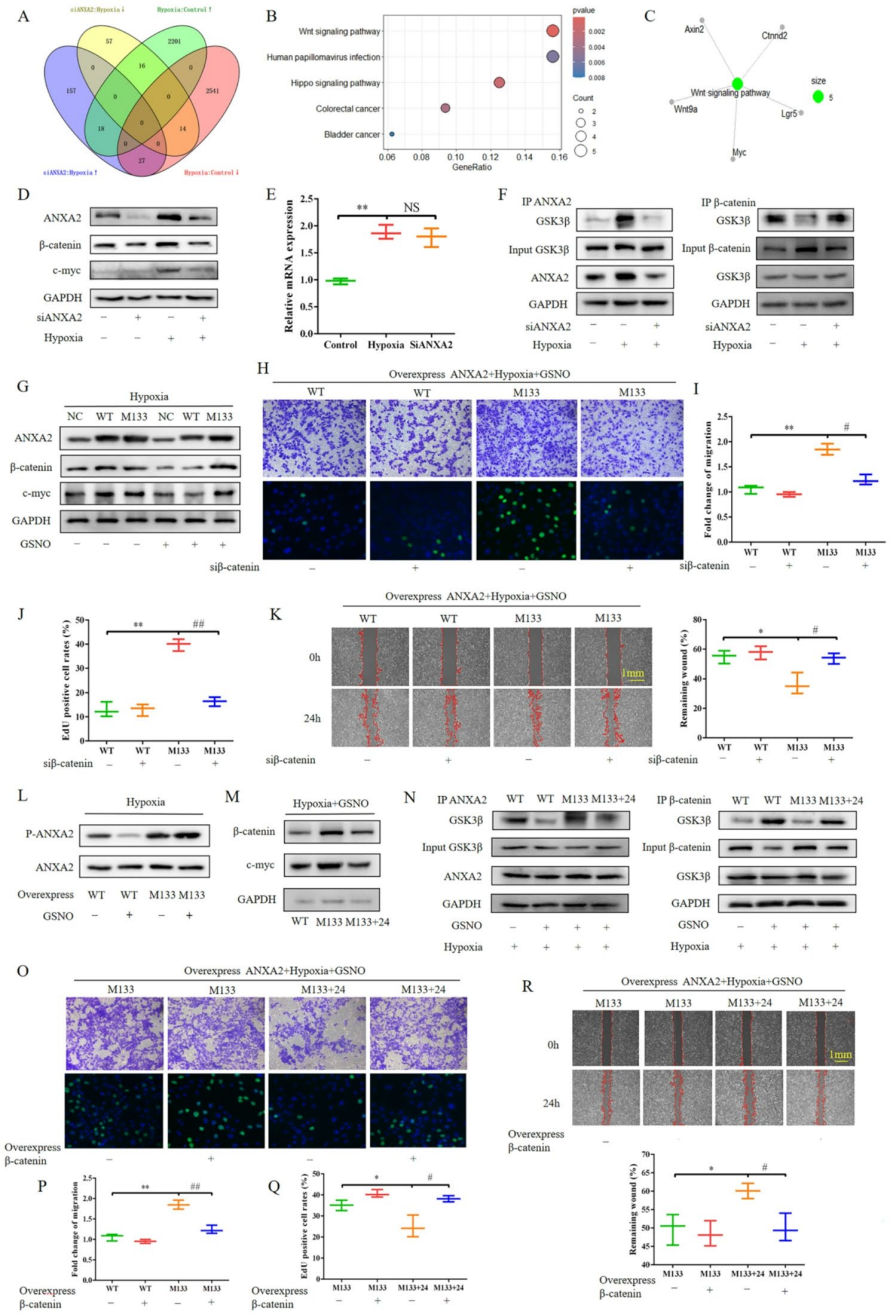
nitrosylation of Cys133 of ANXA2 in PASMCs inhibited hypoxia-induced WNT pathway activation. **A** Venn diagram of 43 genes with opposite changes in expression. **B** Top 5 KEGG pathways of the 43 differentially expressed genes. **C** Gene-Concept Network of the WNT signaling pathway. **D**-**F** Human PASMCs were treated with siANXA2 for 24 h, followed by exposure to hypoxia for 24 h. The protein expression levels of ANXA2, β-catenin, c-myc, and GAPDH in cells were measured by Western blotting (**D**). The mRNA expression levels of β-catenin were measured by PCR (**E**). Coimmunoprecipitation experiments were performed to assess the binding of GSK3β, ANXA2, and β-catenin (**F**). **G**-**K** Overexpression of wild-type and Cys133-mutant ANXA2 in rat PASMCs, followed by treatment with GSNO for 2 h under 24-hour exposure to hypoxia. The protein expression levels of ANXA2, β-catenin, c-myc, and GAPDH in cells were measured by Western blotting (**G**). Cell proliferation and migration were determined by Transwell (up of **H**, scale bar = 200 μm), EdU incorporation assays (drown of **H**, scale bar = 50 μm) and wound healing (**K**, scale bar = 400 μm). **I** Statistical analysis of the fold change of migration. **J** Statistical analysis of the number of EdU-positive cells. **L** The levels of ANXA2 phosphorylation at Tyr24 were measured by Western blotting. (M-R) ANXA2 was overexpressed without mutation, with Cys133 mutation, or with simultaneous mutation of Cys133 and Tyr24 in rat PASMCs, and then, the cells were treated with GSNO for 2 h under 24 h of hypoxia. The protein expression levels of β-catenin, c-myc, and GAPDH in cells were measured by Western blotting (**M**). Coimmunoprecipitation experiments were performed to detect the binding of GSK3β, ANXA2, and β-catenin (**N**). Cell proliferation and migration were determined by Transwell (up of **O**, scale bar = 200 μm), EdU incorporation assays (drown of **O**, scale bar = 50 μm) and wound healing (**R**, scale bar = 400 μm). **P** Statistical analysis of the fold change of migration. **Q** Statistical analysis of the number of EdU-positive cells. The results are presented as the means ± SDs; *, *p* < 0.05; **, *p* < 0.01; ^#^, *p* < 0.05; ^##^, *p* < 0.01

To investigate the molecular signaling mechanism by which ANXA2 participates in PASMC proliferation and migration after exposure to hypoxic conditions, rat PASMCs were subjected to hypoxia for 24 h after transfection with ANXA2 siRNA or control siRNA, and then, RNA was isolated and RNA-seq analysis was performed. A total of 202 upregulated genes and 87 downregulated genes were identified. We merged these genes with the differentially expressed genes that were identified in rat PASMCs that were subjected to hypoxia in the GSE72181 databases [28], and a total of 43 genes with opposite changes in expression were identified (Fig. 4A). KEGG enrichment analysis of the 43 genes revealed that ANXA2 may regulate the WNT signaling pathway (Fig. 4B). The gene-concept network revealed five differentially expressed genes related to the WNT signaling pathway (Fig. 4C). We subsequently investigated the effects of siANXA2 on the WNT pathway under hypoxic conditions. siANXA2 inhibited the hypoxia-induced upregulation of β-catenin and c-Myc in human PASMCs (Fig. 4D). Because ANXA2 downregulation does not affect β-catenin mRNA expression under hypoxic conditions (Fig. 4E), GSK3β-mediated β-catenin ubiquitination is an important degradation pathway. Therefore, we detected the interaction between GSK3β and ANXA2, as well as β-catenin, and found that ANXA2 knockdown significantly inhibited the hypoxia-induced interaction between GSK3β and ANXA2, whereas the interaction between GSK3β and β-catenin was increased (Fig. 4F). These findings indicate that the interaction between ANXA2 and GSK3β reduces the degradation of β-catenin and activates the WNT pathway. However, the Cys133 mutation reversed the inhibitory effect of GSNO on the hypoxia-mediated upregulation of β-catenin and c-Myc (Fig. 4G). When interfering with β-catenin, the excessive proliferation and migration of PASMCs induced by Cys133 mutation under hypoxia and GSNO stimulation were inhibited (Fig. 4H-K).

Since phosphorylation at Tyr24 plays an important role in ANXA2 membrane and nuclear translocation, we detected the level of phosphorylated ANXA2 in PASMCs that were treated with GSNO under hypoxic conditions. Cys133 mutation reversed the decrease in the GSNO-induced phosphorylation of ANXA2 (Fig. 4L). Moreover, compared with the wild-type sequence, the Cys133-mutant sequence did not affect the level of Tyr24 phosphorylation in human PASMCs that were untreated or cultured under normoxic conditions for 24 h (Figure S3). Moreover, compared with Cys133 mutation, Tyr24 mutation decreased the expression of β-catenin and c-Myc in PASMCs that were treated with GSNO under hypoxic conditions (Fig. 4M). In addition, simultaneous mutation of the Cys133 and Tyr24 sites reduced the interaction between ANXA2 and GSK3β compared with mutation of the Cys133 site alone, whereas the interaction between GSK3β and β-catenin was increased (Fig. 4N). Overexpression with β-catenin reversed the inhibitory effects of Cys133 and Tyr24 mutation on the proliferation and migration of PASMCs under hypoxia and GSNO stimulation (Fig. 4O-Q). Overall, these results indicated that SNO-Cys133 of ANXA2 inhibited the WNT pathway by suppressing phosphorylation of its own Tyr 24 residue.

### Mutation of Cys133 in ANXA2 attenuated the effect of GSNO on ameliorating PAH

**Fig. 5:**
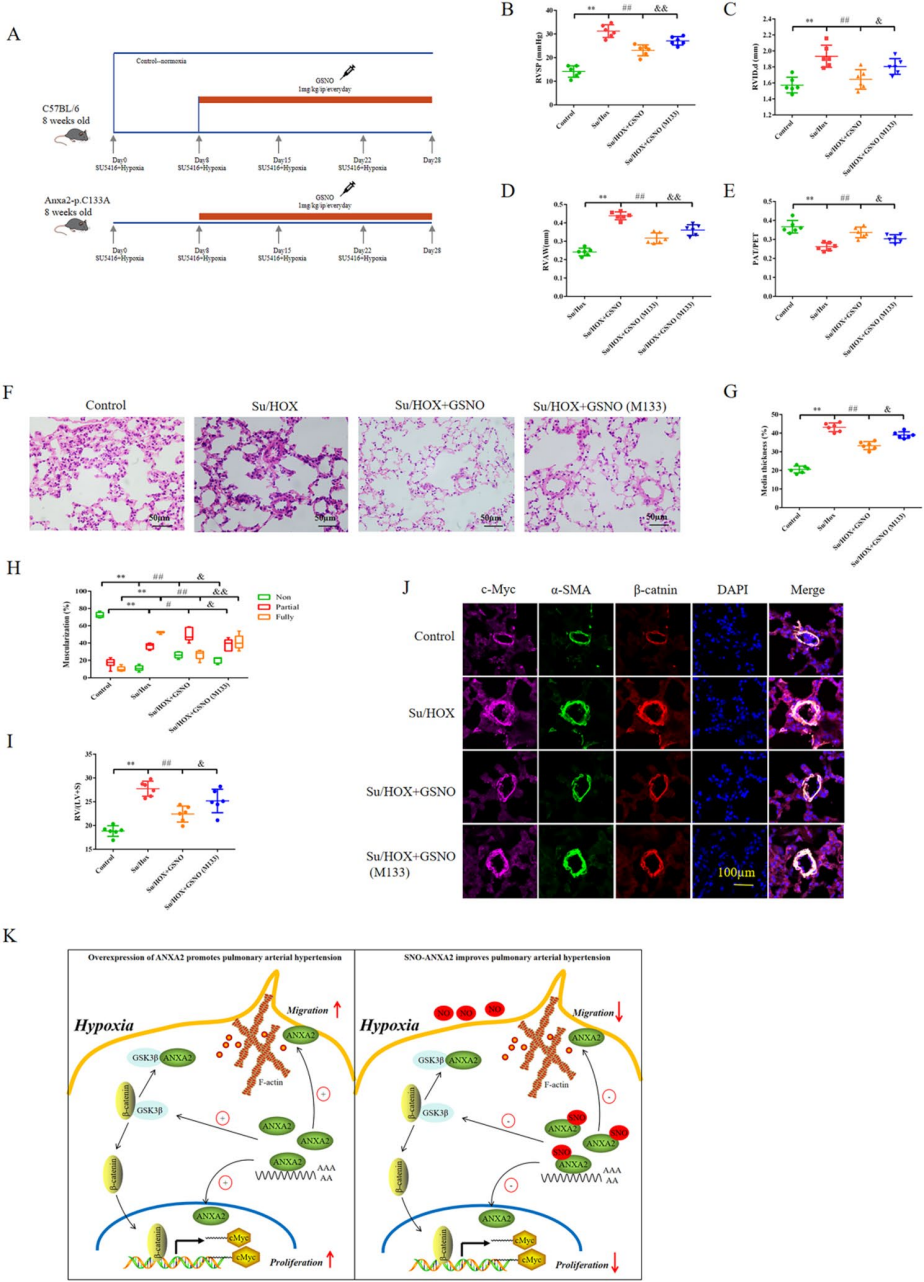
Mutation of Cys133 of ANXA2 attenuated the improvement in pulmonary arterial hypertension caused by GSNO. **A** In vivo experimental design. C57BL/6 mice and mice expressing Cys133-mutant ANXA2 were exposed to hypoxic conditions for 4 weeks and injected with SU5416 once a week. In the second week, PAH model mice were subcutaneously injected with 1 mg/kg GSNO daily. **B** Comparison of the RVSP as measured by a right heart catheter in each group. **C**-**E** After treatment, echocardiograms were recorded. RVAW (**C**), RVID (**D**) and PAT/PET (**E**) were assessed in the control group, Su/Hox group, Su/Hox + GSNO group and Su/Hox + GSNO (M133) group. **F** Representative images of HE staining of distal pulmonary arterioles reflecting pulmonary vascular remodeling. Scale bar = 50 μm. **G**-**I** Comparison of the medial wall thickness (**G**), percentage of muscularization (**H**) and RV/(LV + S) weight (**I**) in each group. **J** Representative confocal immunofluorescence images of pulmonary tissues from each group that were stained with antibodies against β-catenin (red), alpha-SMA (green), and c-Myc (pink) and with DAPI (blue). **K** Schematic diagram summarizing the mechanism of SNO-Cys133 of ANXA2 regulating proliferation and migration of PASMCs. Scale bar = 100 μm. The results are presented as the means ± SDs; *n* = 6 per group. **, *p* < 0.01; ^#^, *p* < 0.05; ^##^, *p* < 0.01; ^&^, *p* < 0.05; ^&&^, *p* < 0.01

To further validate the effect of SNO-Cys133 of ANXA2 on PAH in vivo, PAH was established in C57BL/6 mice and mice expressing Cys133-mutant ANXA2, and these mice were treated with GSNO (Fig. 5A). Although GSNO decreased the RVSP in PAH model mice induced by Su/Hox, mutation of Cys133 increased the RVSP compared with that in the Su/Hox + GSNO group (Fig. 5B). Additionally, Cys133 mutation reversed the effect GSNO on reducing the right ventricular internal diameter (RVID) and RVAW according to echocardiography (Fig. 5C-D), and the PAT: PET ratio was decreased in the Cys133 mutation group compared with the Su/Hox + GSNO group (Fig. 5E). Hematoxylin‒eosin (HE) staining of paraffin-embedded lung sections revealed that GSNO alleviated pulmonary vascular remodeling compared with Su/Hox, whereas the Cys133 mutation exacerbated pulmonary vascular remodeling (Fig. 5F). The GSNO-induced improvement in media wall thickness, muscularization and right ventricle hypertrophy of the distal pulmonary arteries was also reversed by the Cys133 mutation (Fig. 5G-I). Furthermore, IF staining revealed that GSNO downregulated β-catenin and c-Myc expression in the smooth muscle layer of pulmonary arteries, whereas compared with the Su/Hox mutation, the Cys133 mutation upregulated β-catenin and c-Myc expression. These results demonstrated that SNO-Cys133 of ANXA2 alleviates pulmonary vascular and right ventricular remodeling.

## Discussion

Increasing attention has been given to the excessive PASMC proliferation and migration that is observed during PAH pathogenesis, and this study aimed to describe the role of ANXA2 and its S-nitrosylation-mediated signaling in this cellular behavioral change. This novel finding was based on the following observations: (1) ANXA2 promotes excessive PASMC proliferation and migration by translocating to the membrane and nucleus and activating the WNT pathway; (2) in vivo, inhibiting ANXA2 activity or downregulating the high ANXA2 expression in PASMCs alleviates pulmonary vascular remodeling in experimental PAH animal models; (3) SNO-Cys133 of ANXA2, which inhibits hypoxia-mediated PASMC proliferation and migration by regulating the subcellular localization of ANXA2 in cells, was reported for the first time; (4) SNO-Cys133 of ANXA2 regulates the WNT pathway by inhibiting the phosphorylation of ANXA2 at Tyr24; and (5) in vivo, SNO-Cys133 of ANXA2 ameliorates pulmonary vascular remodeling and RV function in experimental PAH animal models (Fig. 5K).

Several studies have indicated that ANXA2 expression in PASMCs is increased in animal models of hepatopulmonary syndrome [15, 16]. We confirmed the upregulation of ANXA2 expression in PASMCs from PAH model mice and in PASMCs exposed to hypoxic conditions. Consistent with previous studies, inhibiting ANXA2 reduces the excessive proliferation and migration of cells caused by hypoxia [29, 30]. ANXA2-mediated cell migration is associated with the regulation of F-actin remodeling to promote pseudopodia formation [31, 32]. In this study, hypoxia led to increased ANXA2 membrane localization, which is consistent with the formation of a complex between ANXA2 and S100A11 on the cell membrane, which in turn regulates pseudopodia formation [31]. Owing to the increased expression of ANXA2, in addition to its translocation to the cell membrane, the nuclear localization of ANXA2 also increases. Studies have shown that the nuclear localization of ANXA2 promotes cell proliferation through various signaling mechanisms, such as activation of the Hippo pathway or the signal transducer and activator of transcription 3 (STAT3) signaling pathway [33, 34]. However, the mechanism by which ANXA2 nuclear translocation is mediated by hypoxia, resulting in PASMC proliferation, requires further exploration.

KEGG enrichment analysis of the RNA sequencing and subsequent signal mechanism verification results revealed that ANXA2 activates the WNT pathway in PASMCs that are exposed to hypoxic conditions. WNT/β-catenin signaling is an important regulator of PAH occurrence and development [35, 36]. Recently, studies have shown that ANXA2 activates the WNT pathway to promote cell differentiation, proliferation, and migration [22, 23, 37]. ANXA2 binds to GSK3β and suppresses the ubiquitination-mediated degradation of β-catenin [37, 38]. Here, we found that SNO-Cys133 of ANXA2 reduces the binding of GSK3β to ANXA2, providing a potential mechanism for the inhibition of the WNT signaling pathway, which is a key pathway in PASMC proliferation and migration.

NO-induced nitrated products play major roles in modulating vascular tone and remodeling in pulmonary circulation [27]. The NO signaling pathway has become one of the important signaling mechanisms that is used in the discovery of drugs for treating PAH [39]. SNO, which is the oxidative modification of cysteine by NO to form protein S-nitrosothiols, regulates various cellular biological behaviors related to NO and is associated with diverse pathophysiological mechanisms [40]. Our research revealed that the signaling pathway mediated by SNO-Cys133 of ANXA2 may be another important mechanism by which NO ameliorates PAH. One of the reasons is that SNO-Cys133 inhibits ANXA2 membrane and nuclear localization. Another reason is that the GSNO-induced SNO of Cys133 suppresses the phosphorylation of ANXA2 at Tyr24. Previous studies revealed that phosphorylation of ANXA2 at Tyr24 leads to its translocation into the membrane and nucleus and enhances tumor cell migration and proliferation [10, 30]. In our study, the phosphorylation of Try24 also affected the binding between SNO-Cys133 of ANXA2 and GSK3β. Notably, ANXA2 contains four cysteine residues (Cys9, Cys133, Cys262, and Cys335). After the mutation of Cys133, SNO-ANXA2 can still be detected by Western blotting without any treatment. This finding suggests that other sites also undergo SNO.

In summary, our present work provides both in vitro and in vivo evidence that ANXA2 contributes to PASMC proliferation and migration during PAH development through its intracellular redistribution and WNT pathway activation. More importantly, SNO-Cys133 of ANXA2 suppresses the relocation of ANXA2 and activates the WNT pathway to improve pulmonary vascular remodeling in pulmonary hypertension. Thus, our findings not only provide new insights into the signaling mechanism underlying PASMC proliferation and migration in the context of PAH but also may have important implications for the development of novel strategies for PAH treatment that target the SNO-Cys133 of ANXA2.

## Supplementary Information

Supplementary Material 1.

Supplementary Material 2.

Supplementary Material 3.

Supplementary Material 4.

## Data Availability

The data that support the findings of this study are available from the corresponding author upon reasonable request.
