# Supplementary material for: S- nitrosylation of Annexin A2 at Cys133 ameliorates pulmonary arterial hypertension by inhibiting the WNT/β-catenin pathway
Source: Respir Res. 2026 Mar 18;27:217. doi: 10.1186/s12931-025-03483-4 (PMC13224607; doi:10.1186/s12931-025-03483-4)
Supplement: Supplementary file 1 — Supplementary Material 1. [file 12931_2025_3483_MOESM1_ESM.pdf]

Figure1 A

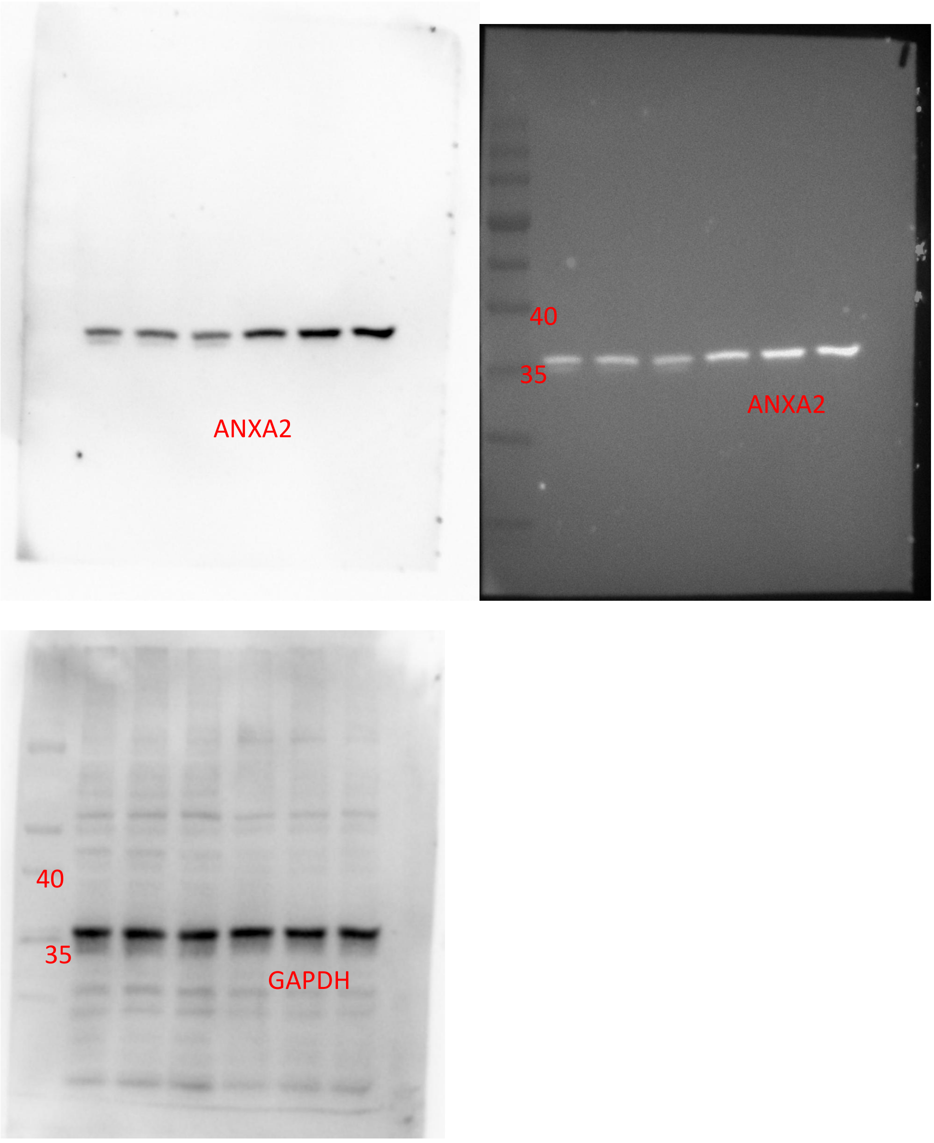

Figure3 B

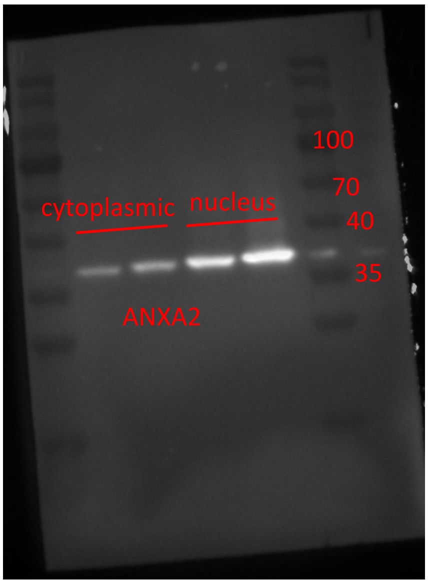
cytoplasmic
nucleus
100
70
40
35
ANXA2

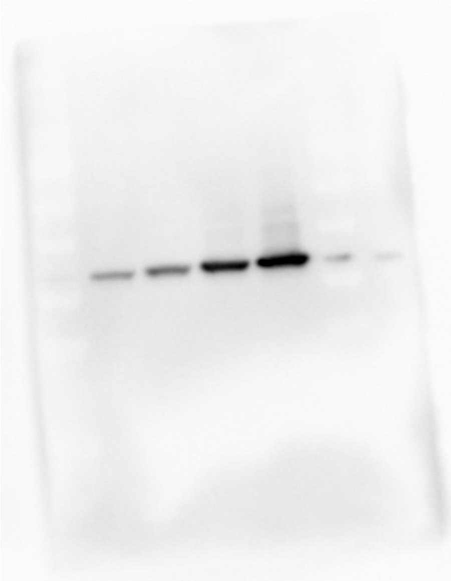

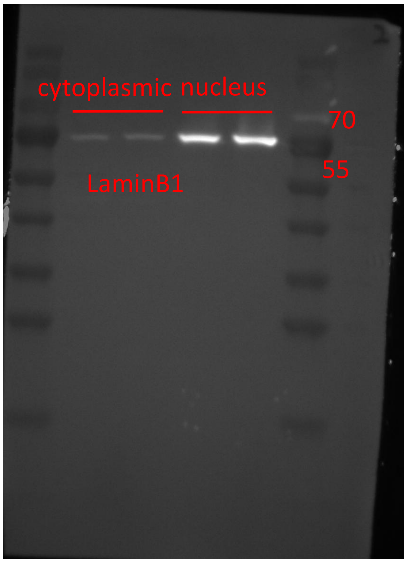
cytoplasmic
nucleus
70
55
LaminB1

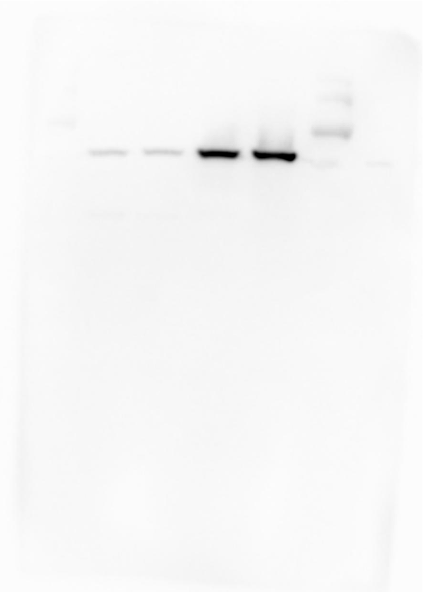

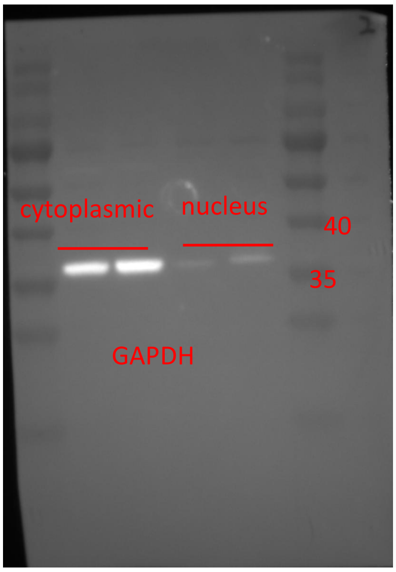
cytoplasmic
nucleus
40
35
GAPDH

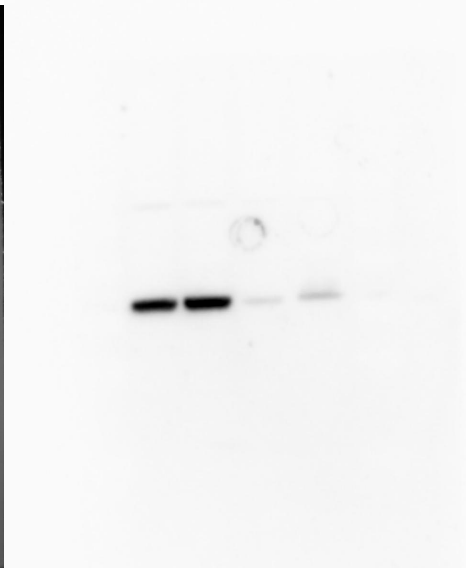

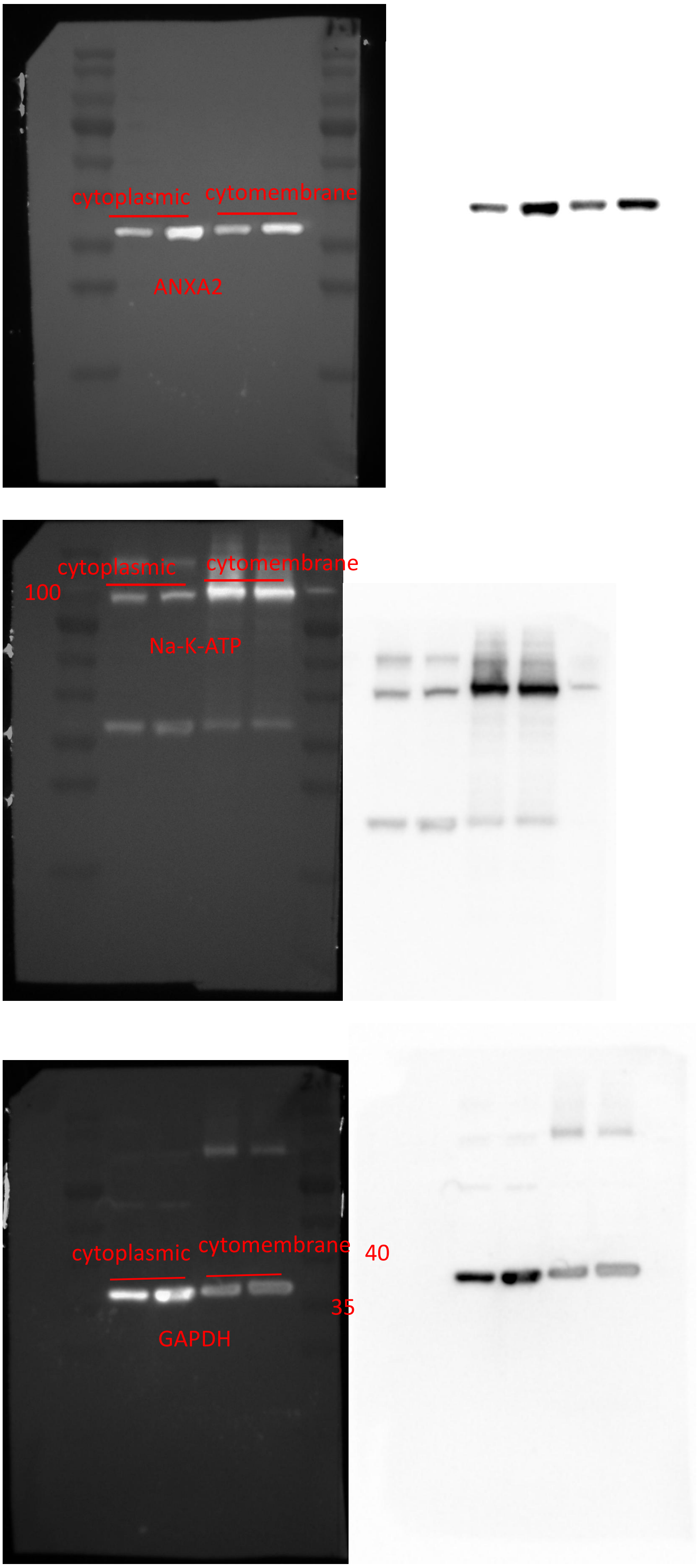
cytoplasmic
cytomembrane
ANXA2
cytoplasmic
cytomembrane
100
Na-K-ATP
cytoplasmic
cytomembrane
40
35
GAPDH
Figure3 C

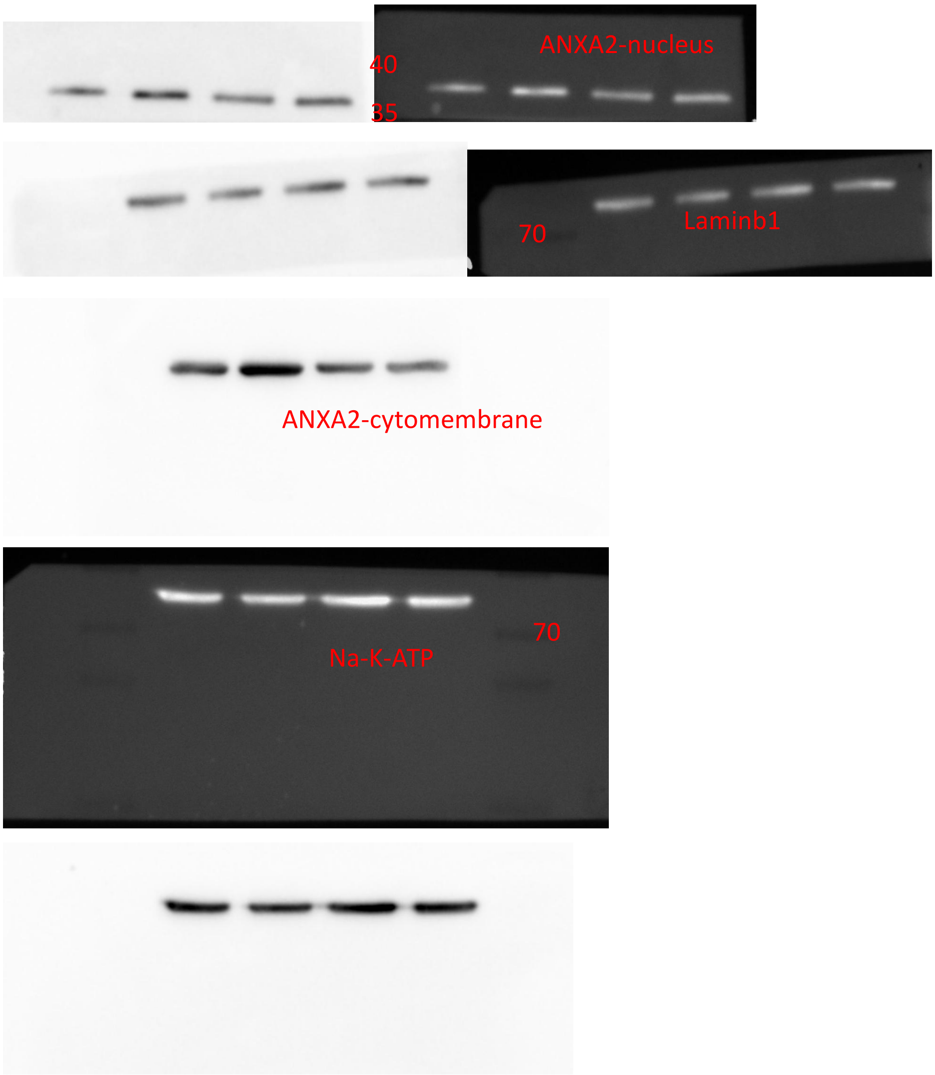
ANXA2-nucleus
40
35
Laminb1
70
ANXA2-cytomembrane
Na-K-ATP
70

Figure3 D

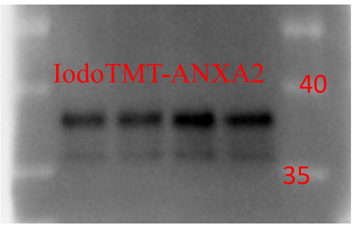
IodoTMT-ANXA2
40
35

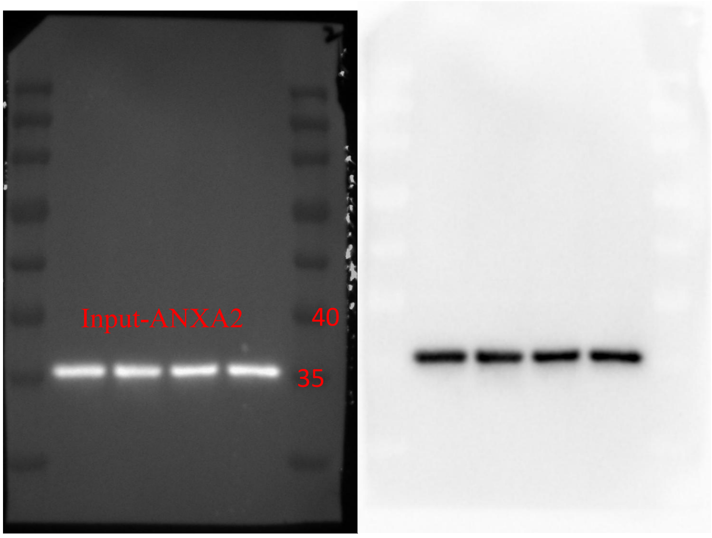

Figure3 F

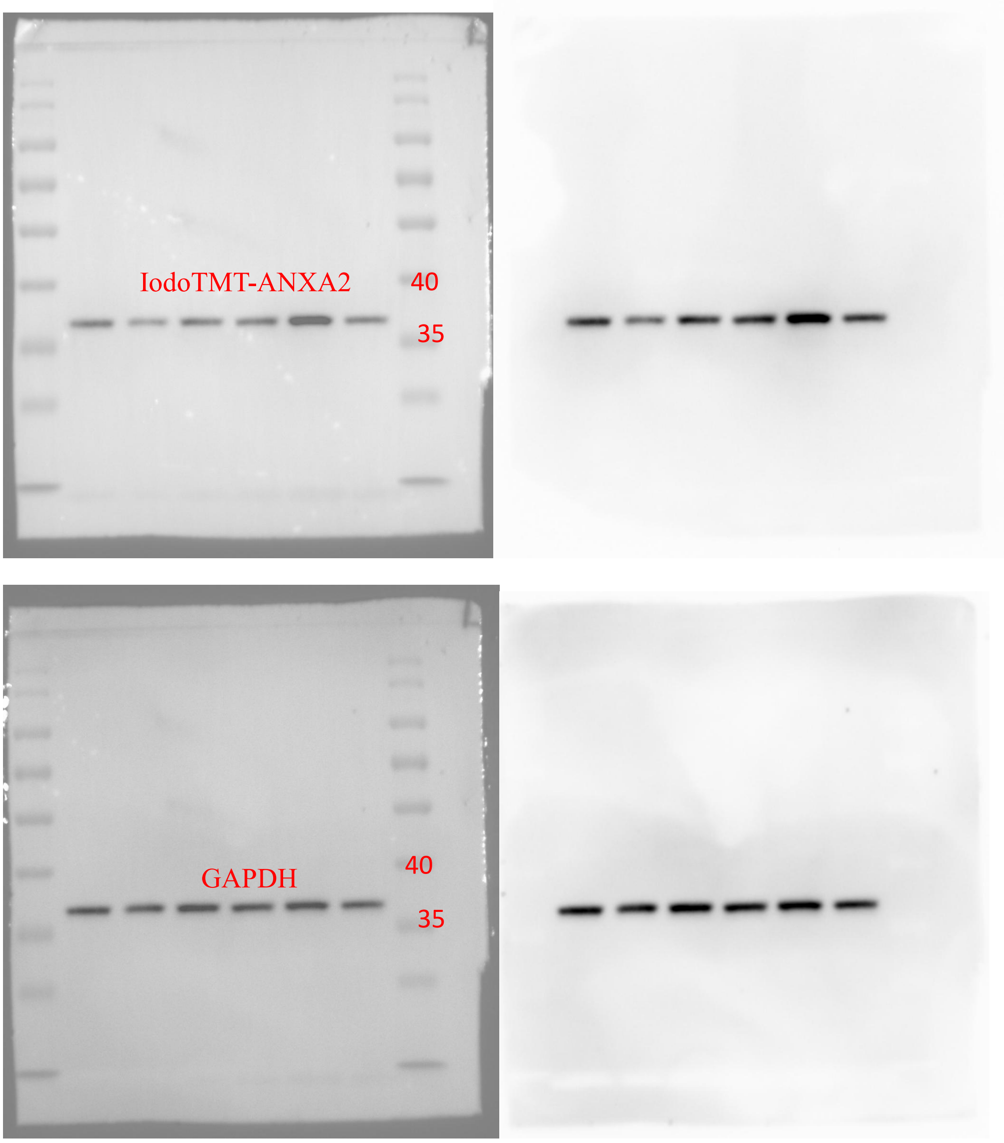

Figure4 D

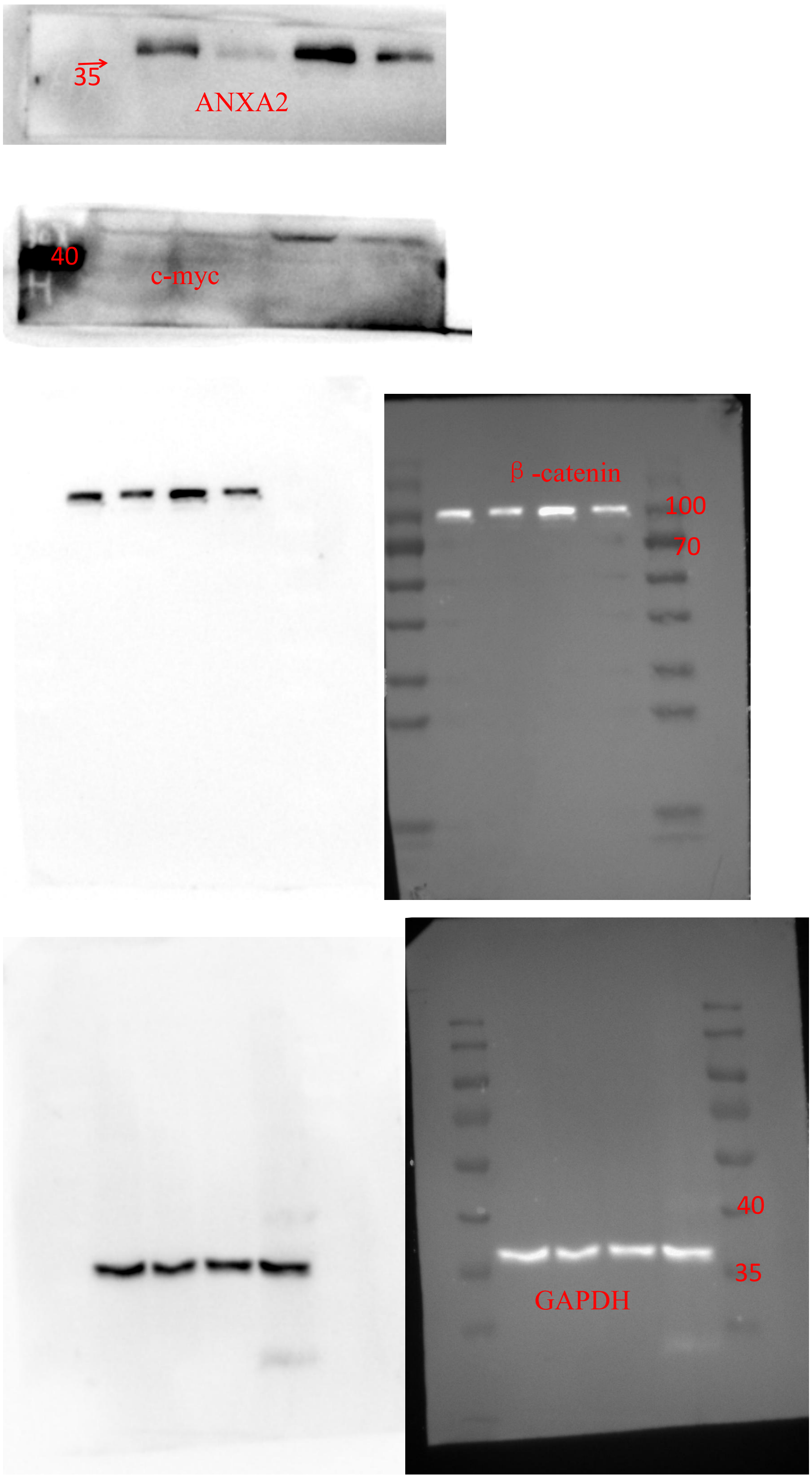

Figure4 F
Right

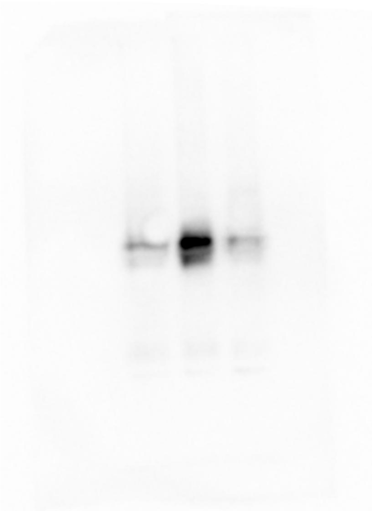

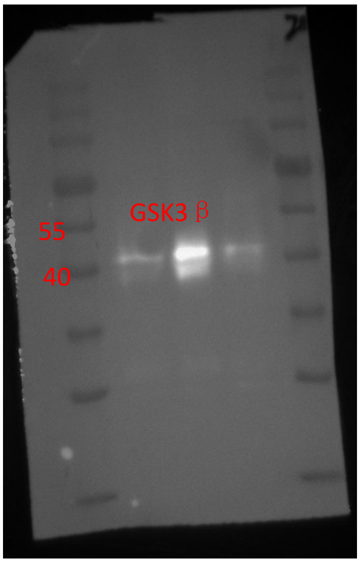
GSK3 β
55
40

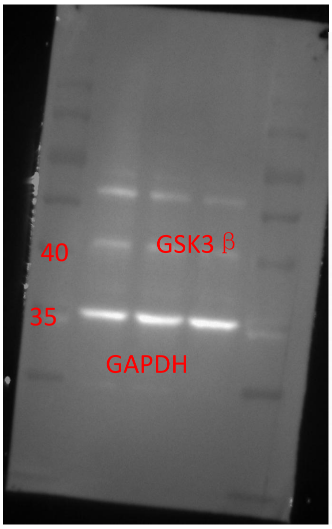
40
GSK3 β
35
GAPDH

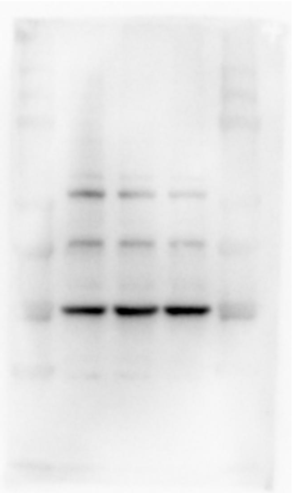

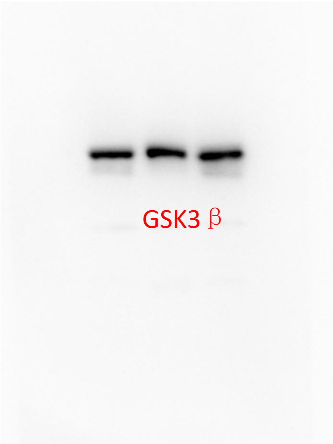
GSK3 β

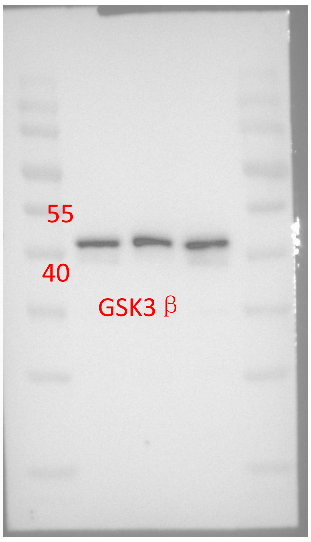
55
40
GSK3 β

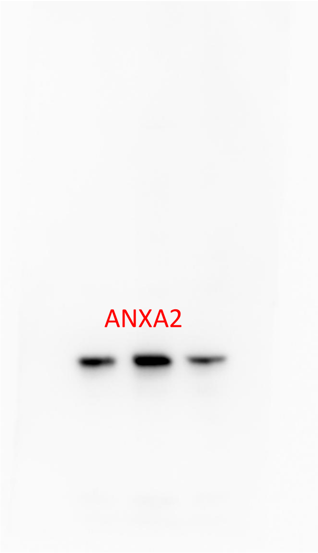

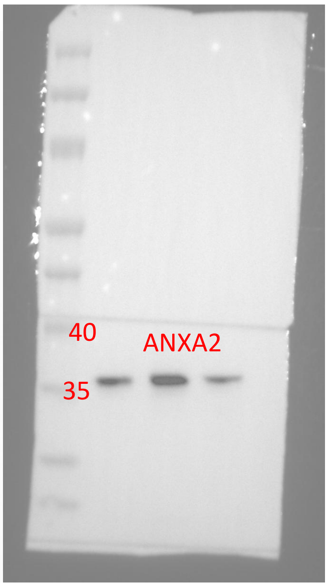

Left

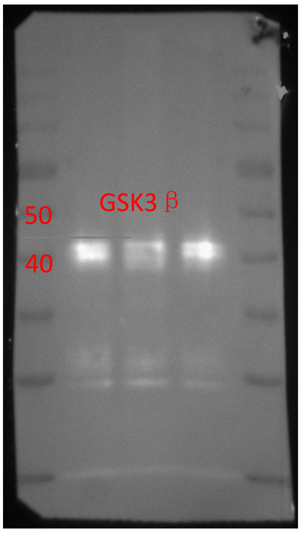

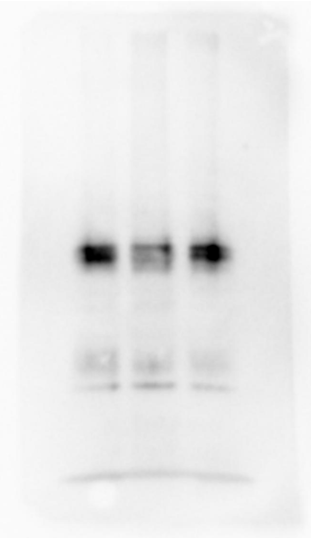

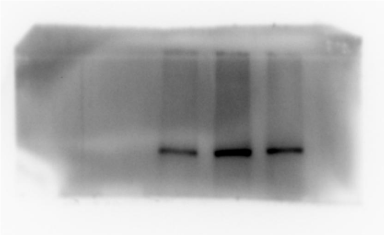

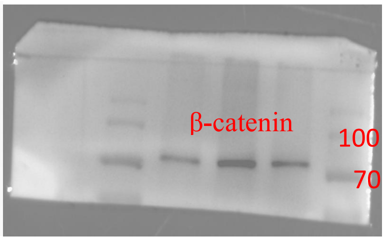

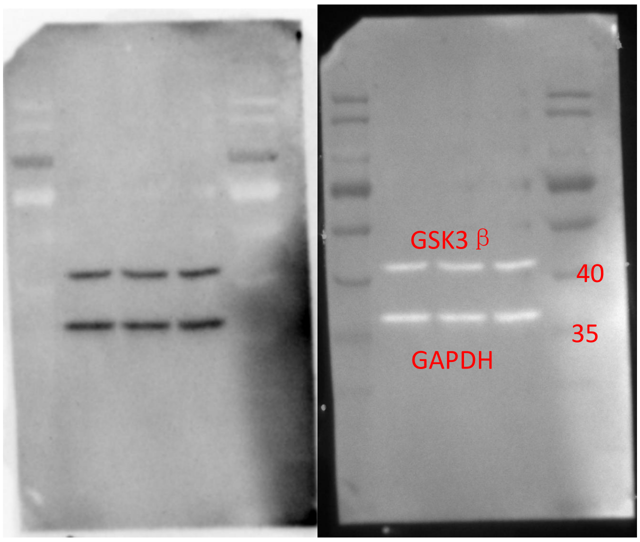

Figur4 G

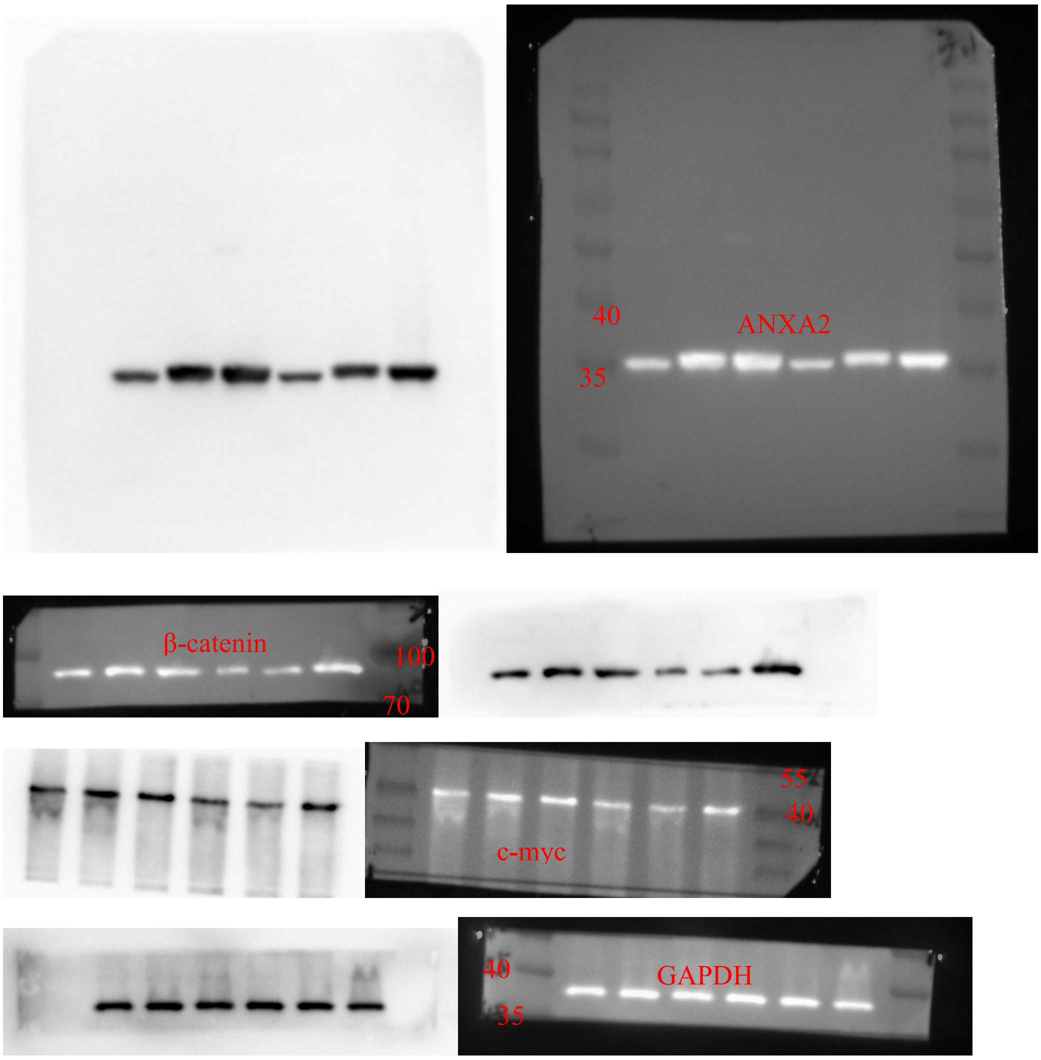

Figure4 H

P-ANXA2

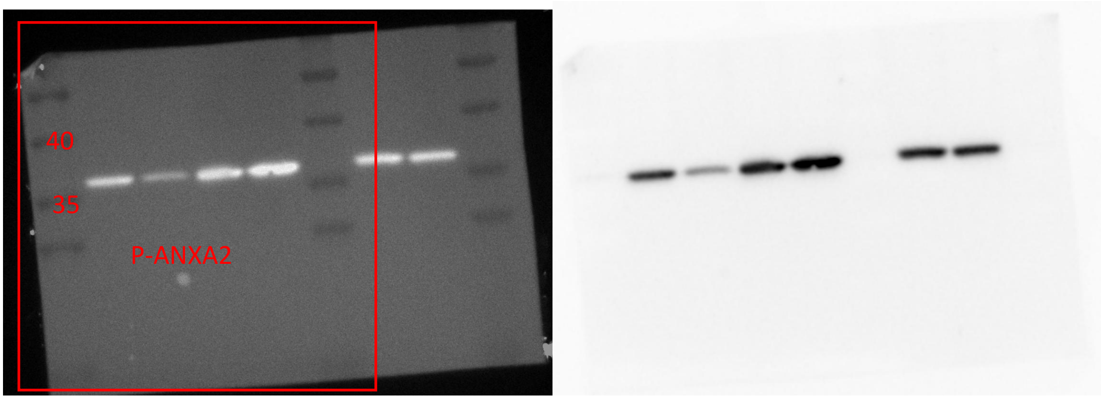

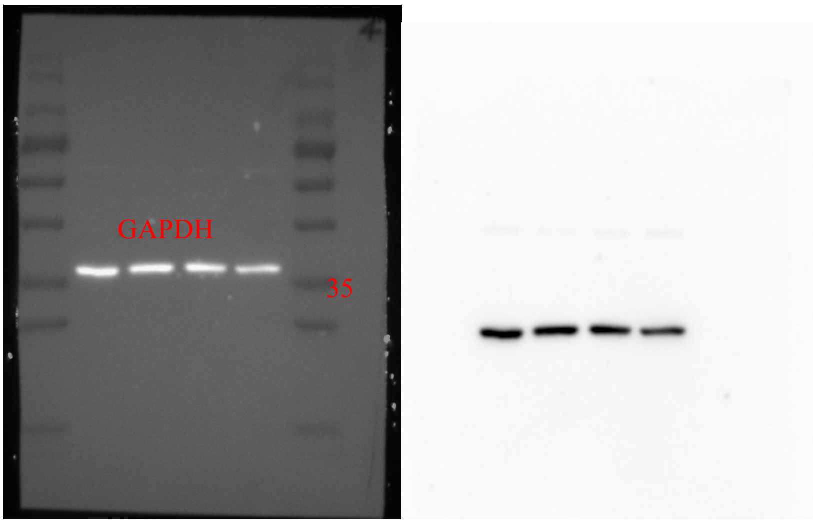

Figure4 I

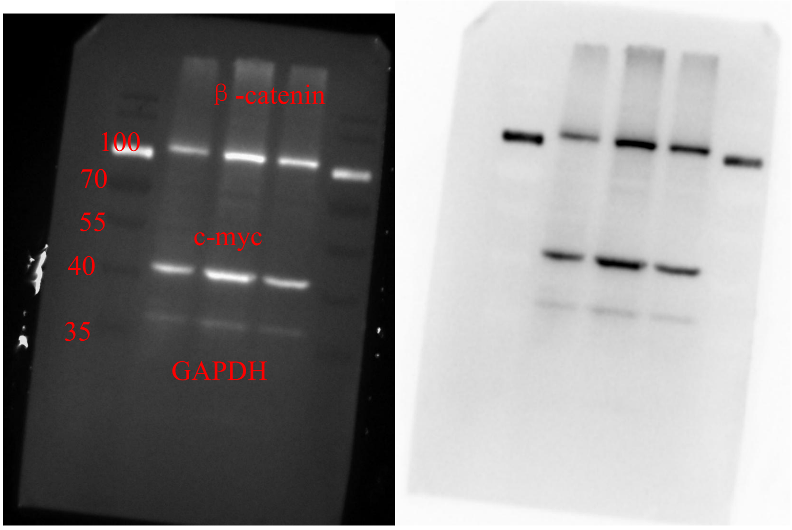

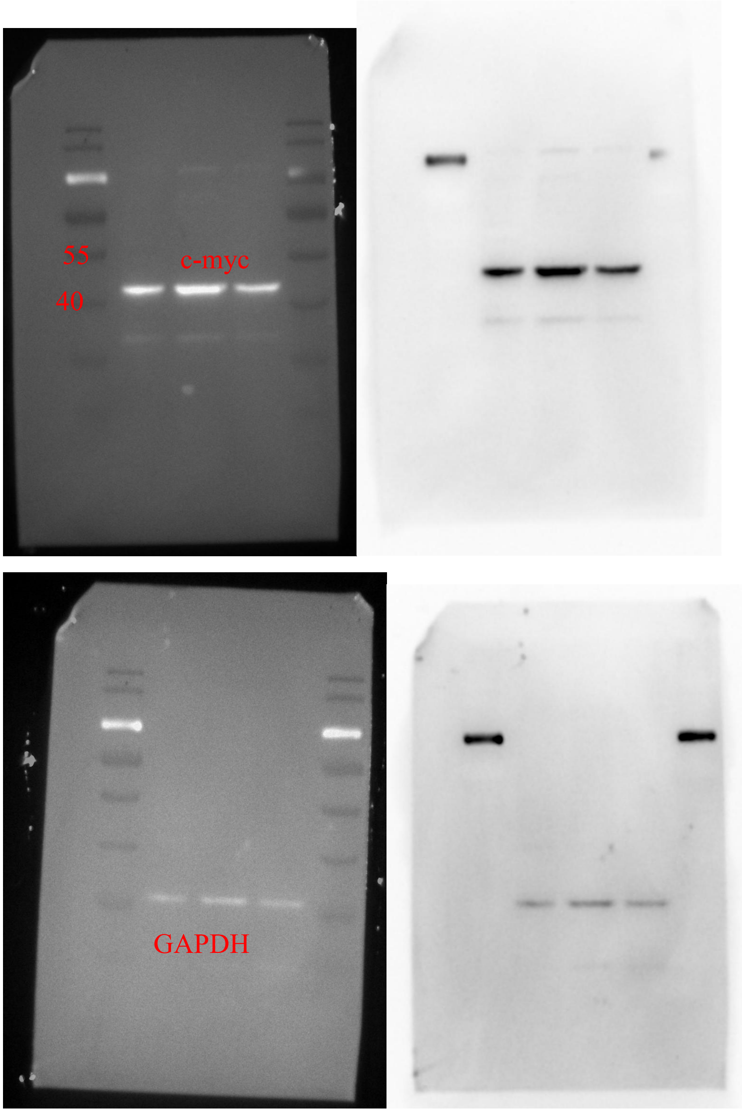

Figure4 J

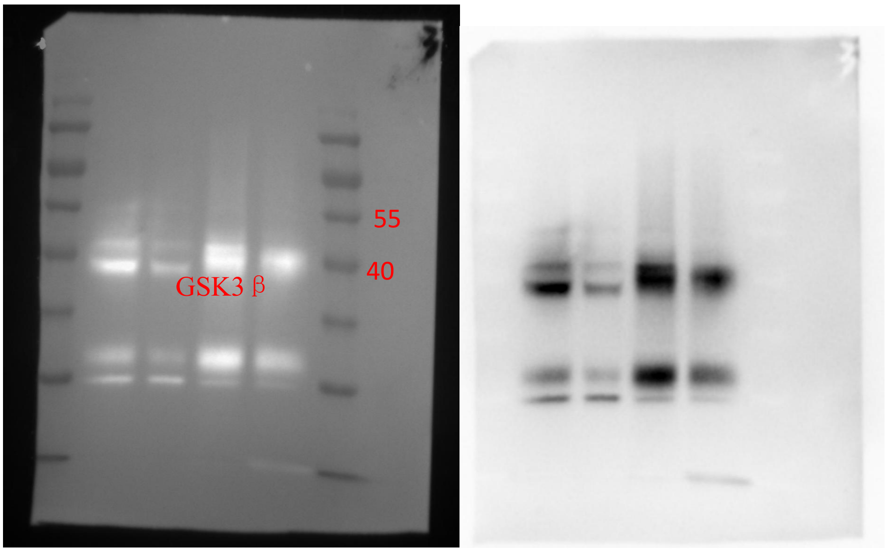

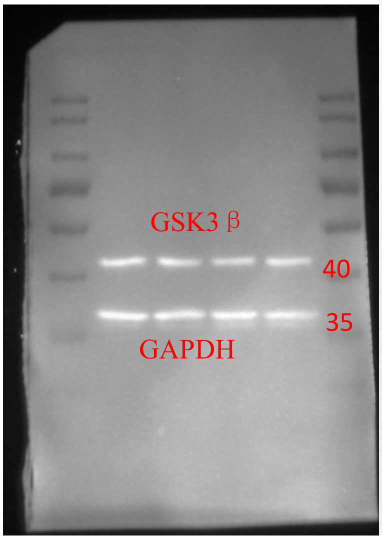
GSK3 β
40
35
GAPDH

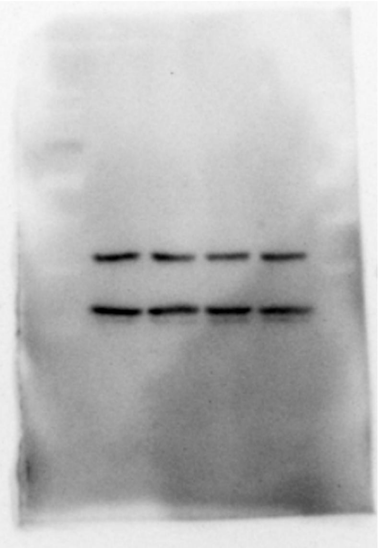

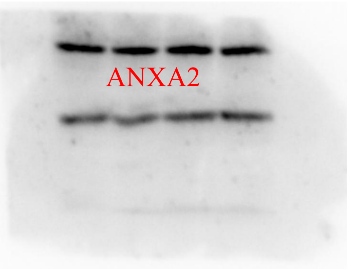
ANXA2

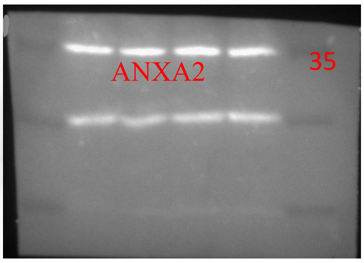
ANXA2
35

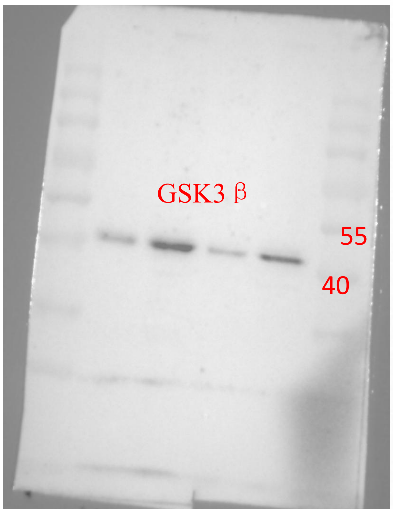
GSK3 β
55
40

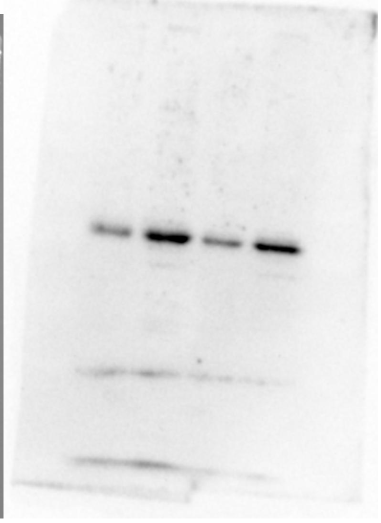

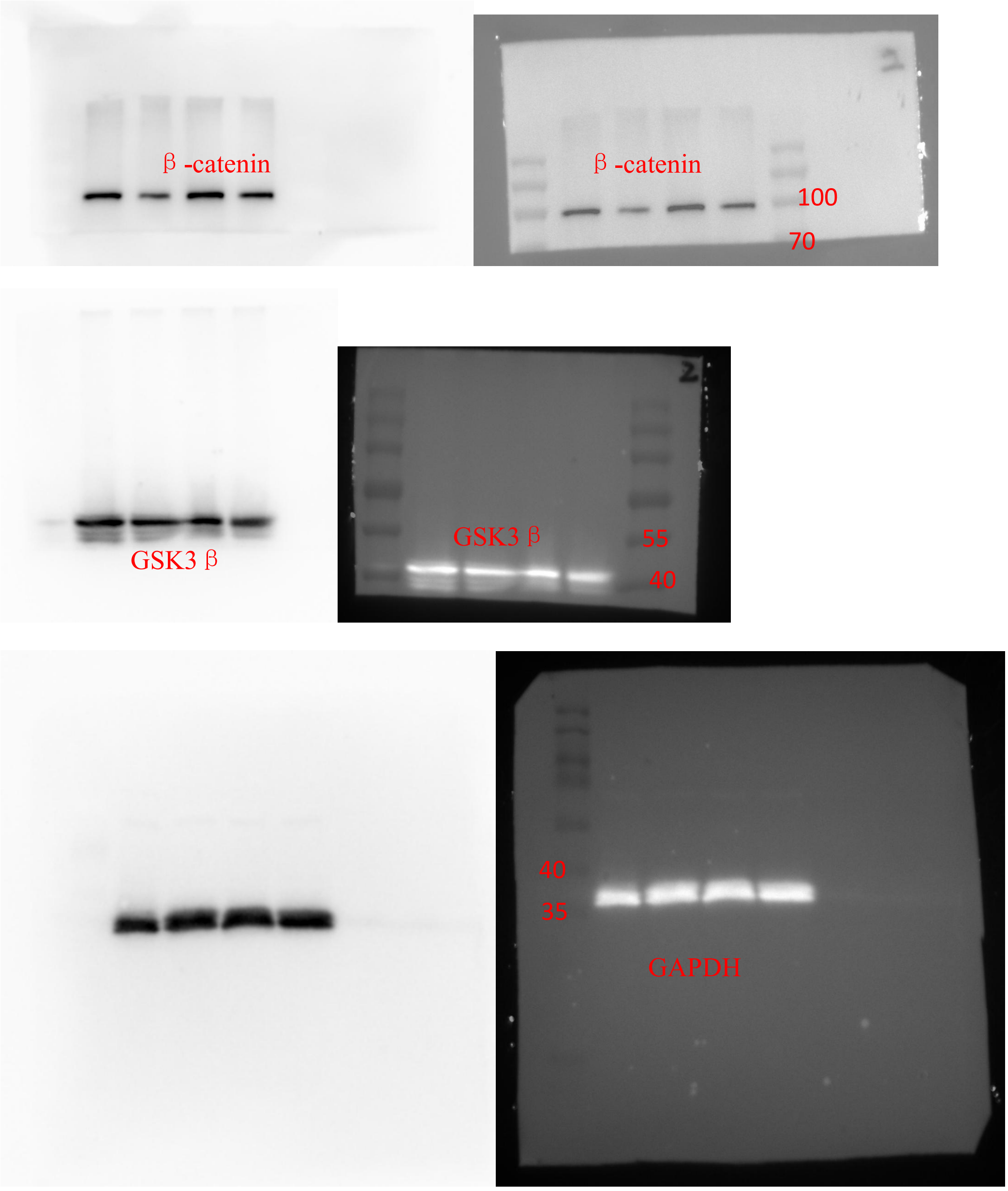
β -catenin
β -catenin
100
70
GSK3 β
GSK3 β
55
40
40
35
GAPDH
