# Supplementary material for: S- nitrosylation of Annexin A2 at Cys133 ameliorates pulmonary arterial hypertension by inhibiting the WNT/β-catenin pathway
Source: Respir Res. 2026 Mar 18;27:217. doi: 10.1186/s12931-025-03483-4 (PMC13224607; doi:10.1186/s12931-025-03483-4)

**Supplement**

Figure S1

Inhibiting ANXA2 suppressed the proliferation and migration of PASMCs from rats with PAH.

PASMCs were pretreated with LCKLSL (1 mM) for 2 hours and then subjected to hypoxic conditions for 24 hours. Cell proliferation and migration were assessed by Transwell assays (A, scale bar = 200 µm), wound healing assays (B, scale bar = 400 µm) and MTT assays (C). The results are presented as the means ± SDs; *, p < 0.05; **, p < 0.01.


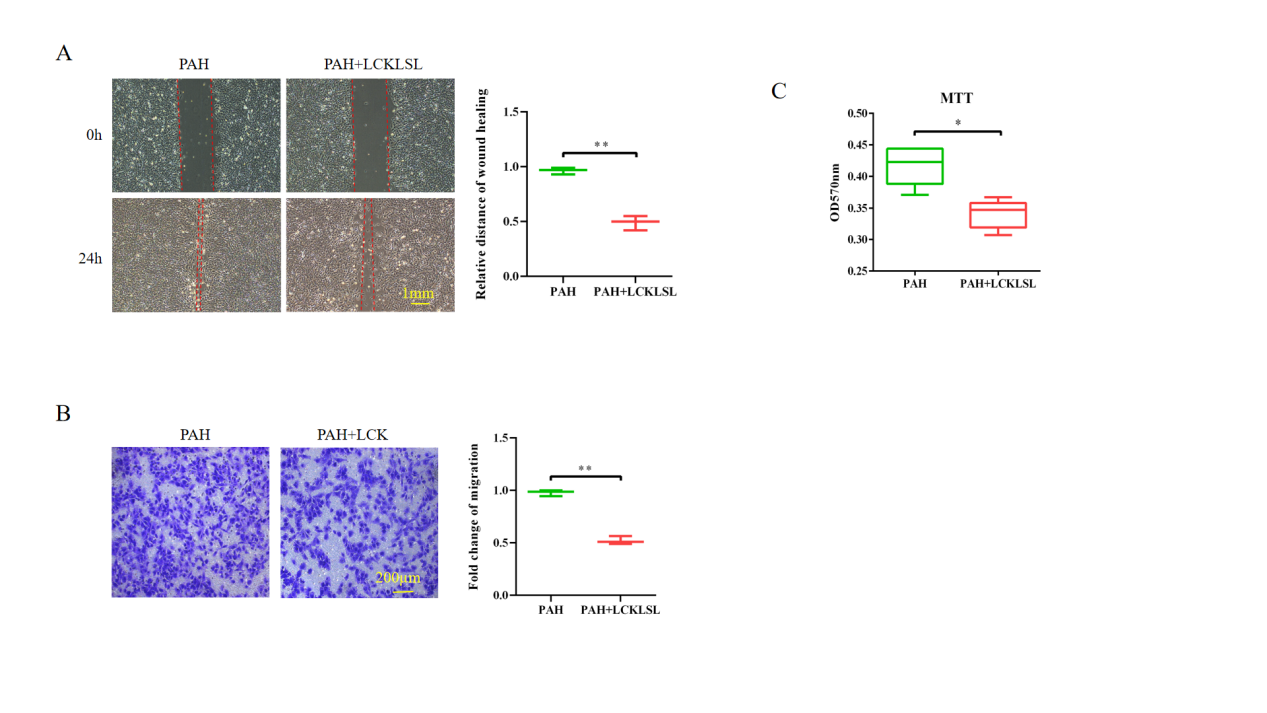


Figure S2

Inhibition of ANXA2 mitigated pulmonary vascular remodeling and improved right ventricular function in experimental with PAH. Male Sprague‒Dawley rats (6 weeks old) were exposed to hypoxic conditions (10% O_2_) for 4 weeks and injected with SU5416 once a week to establish the SU/Hox model. In addition, male Sprague‒Dawley rats (6 weeks old) were subcutaneously injected with a single dose of 60 mg/kg monocrotaline (MCT) to establish the MCT model. From day 15 to day 28 of model establishment, all the rats with experimental PAH continuously received 5 mg/day/kg body weight PX-12 via subcutaneous injection every day. (A) Comparison of pulmonary arterial pressure as measured by a right heart catheter in each group. (B) Representative images of HE-stained distal pulmonary arterioles reflecting pulmonary vascular remodeling. Scale bar = 100 µm. (C-E) Comparison of the medial wall thickness (C), percentage of muscularization (D) and RV/(LV + S) weight (E) in each group. The results are presented as the means ± SDs; n = 6 per group. *, p < 0.05; **, p < 0.01; ^#^, p < 0.05; ^##^, p < 0.01.


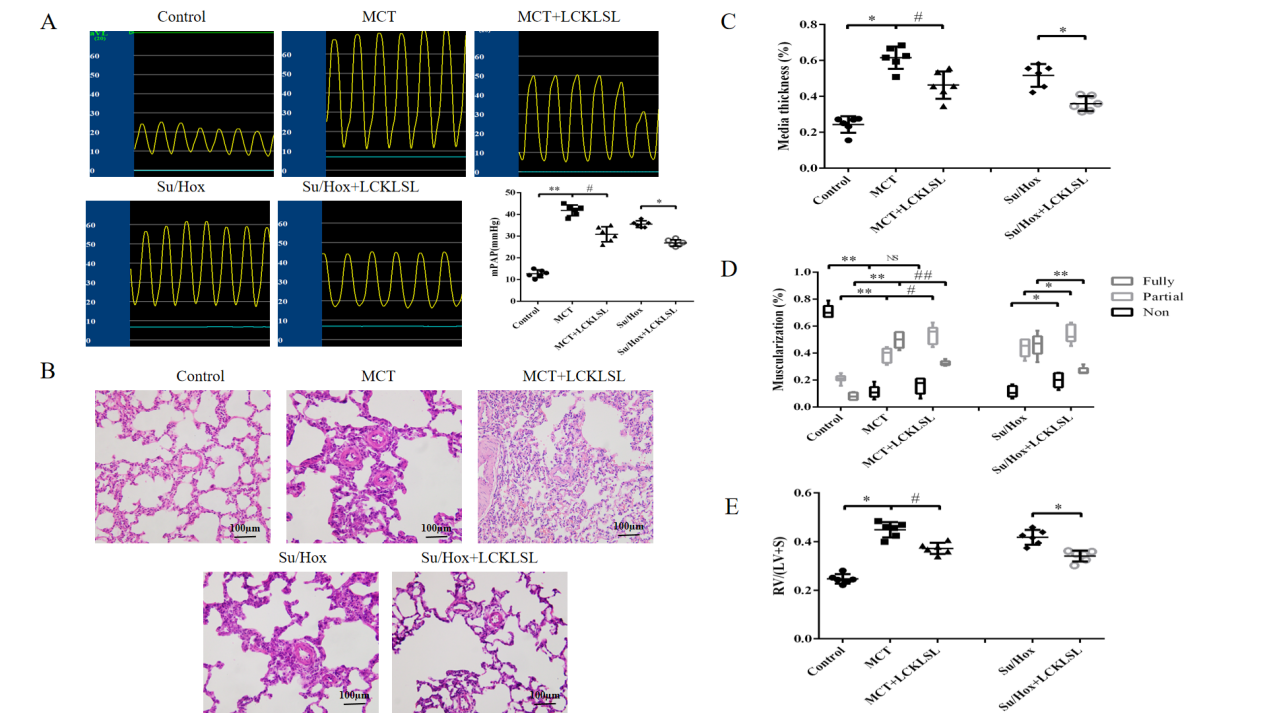


Figure S3

Compared with the wild-type sequence, the Cys133-mutant sequence did not affect the level of phosphorylation at Tyr24 in PASMCs that were untreated or cultured under normoxic conditions.

Wild-type or Cys133-mutated ANXA2 was overexpressed in rat PASMCs, which were then treated with GSNO for 2 hours under 24 hours of exposure to hypoxia. The levels of phosphorylated ANXA2 (P-ANXA2), ANXA2, and GAPDH in cells were measured by Western blotting.


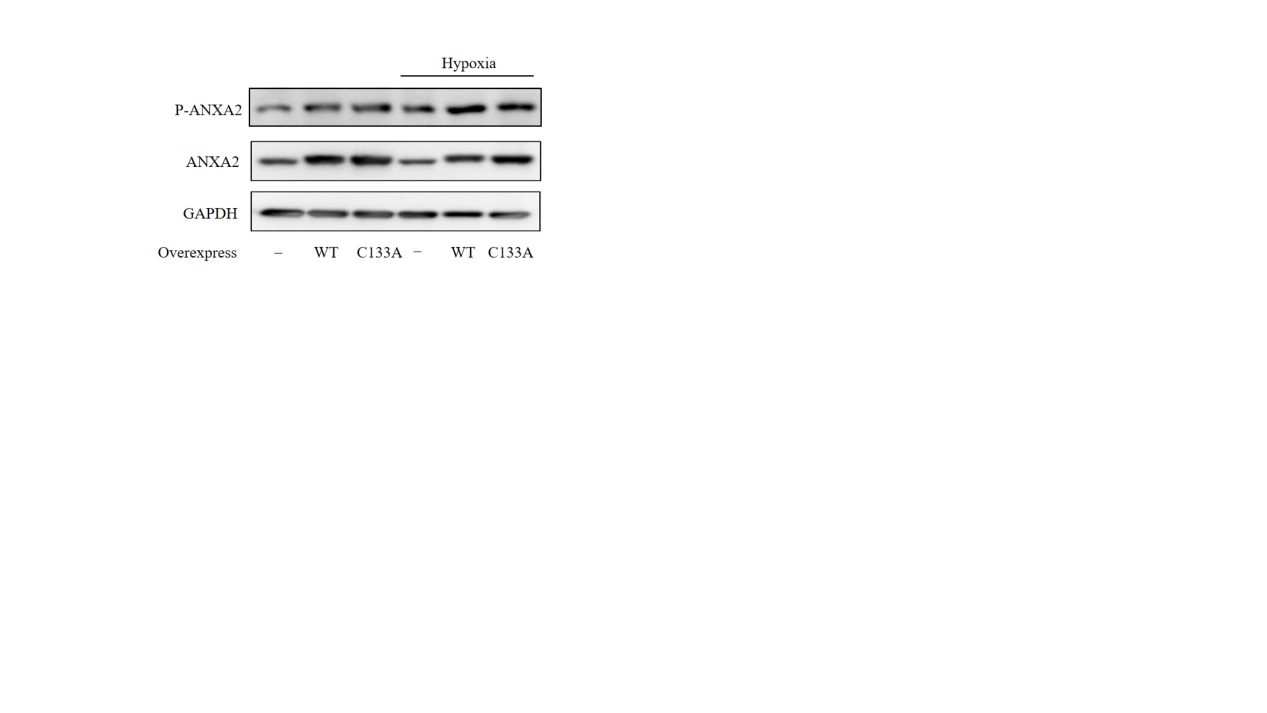

Supplement: Supplementary file 2 — Supplementary Material 2. [file 12931_2025_3483_MOESM2_ESM.docx]
